# Supplementary material for: Nanobody-enabled monitoring of kappa opioid receptor states
Source: Nat Commun. 2020 Mar 2;11:1145. doi: 10.1038/s41467-020-14889-7 (PMC7052193; doi:10.1038/s41467-020-14889-7)
Supplement: Supplementary file 1 — Supplementary Information [file 41467_2020_14889_MOESM1_ESM.pdf]

Supplementary Information

Nanobody-Enabled Monitoring of Kappa Opioid Receptor States

Tao Che, Justin English, Brian E. Krumm, Kuglae Kim, EIs Pardon, Reid HJ. Olsen, Sheng Wang, Shicheng Zhang, Jeffrey F. Diberto, Noah Sciaky, F. Ivy Carroll, Jan Steyaert, Wacker Daniel, Bryan L. Roth.

a

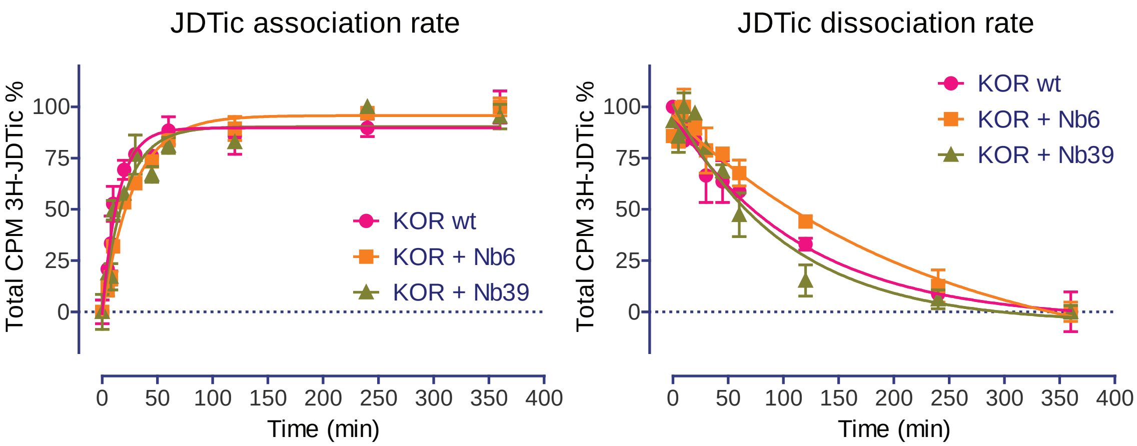

JDTic Dissociation and Association Rates at Wild-Type, Nb6- and Nb39-stabilized KOR.				
Receptor	B_{MAX} ± SEM, CPM	Half life Time, min (k_{off} ± SEM) min^{-1}	k_{on} ± SEM, M^{-1} min^{-1}	K_d, nM (pKd ± SEM)
KOR wt	412 ± 46	125 (0.008 ± 0.002)	$1.31 \times 10^8 \pm 8.55 \times 10^7$	0.065 (10.19 ± 0.23)
KOR + Nb6	468 ± 54	250 (0.004 ± 0.001)	$7.29 \times 10^7 \pm 4.89 \times 10^6$	0.057 (10.24 ± 0.18)
KOR + Nb39	423 ± 59	111 (0.009 ± 0.002)	$7.42 \times 10^8 \pm 8.06 \times 10^7$	0.069 (10.16 ± 0.37)

b

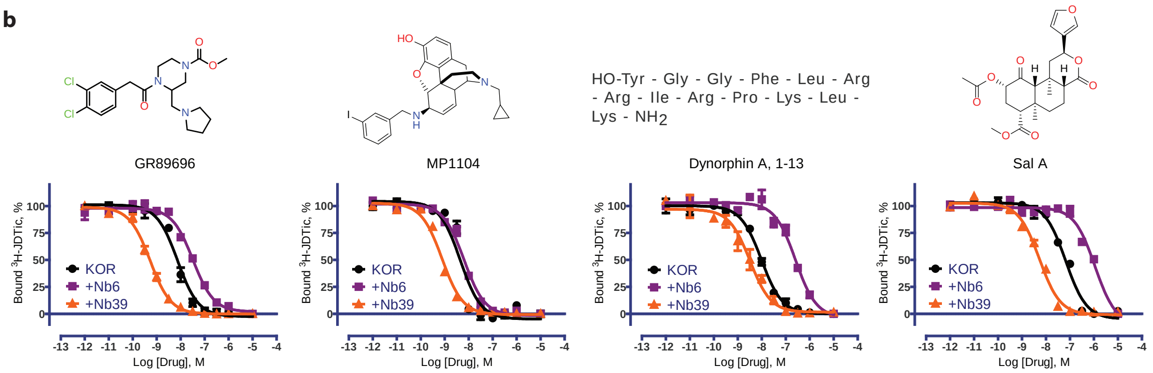

KOR ligands	K_i, nM		
	KOR apo	+ Nb6	+ Nb39
GR89696	1.07 ± 0.13	4.67 ± 0.51*	0.07 ± 0.01*
MP1104	0.64 ± 0.15	0.91 ± 0.10	0.11 ± 0.01*
Dynorphin A, 1-13	2.43 ± 0.20	64.2 ± 8.0*	0.81 ± 0.13
Sal A	9.51 ± 1.19	157 ± 23*	0.76 ± 0.07*

*** p<0.05 vs KOR apo.** Data represent mean Ki in nM for N=3 separate experiments.

Supplementary Figure 1. Differential effects of Nb6 or Nb39 on ligand's binding affinity. (a) The saturation binding kinetics of JDTic were measured in the presence of Nb6 or Nb39. The parameter values were summarized in the table. Data were acquired by association and dissociation kinetic experiments conducted in parallel at room temperature using [^{3}H]-JDTic (concentration range 0.6–1.5 nM). Estimates of k_{off}, k_{on}, and K_d were obtained from three independent experiments performed in duplicate. The residence time was calculated as 1/k_{off}. **(b)** Competition binding studies using ^{3}H-JDTic, the indicated non-labeled competitor, HEK 293T cell membrane expressing human KOR, and 5 µM of Nb6 or Nb39. The value of each binding affinity is summarized in the table. *P < 0.05, F test (N=3, three experiments each done in duplicate). Source data are provided as a Source Data file.

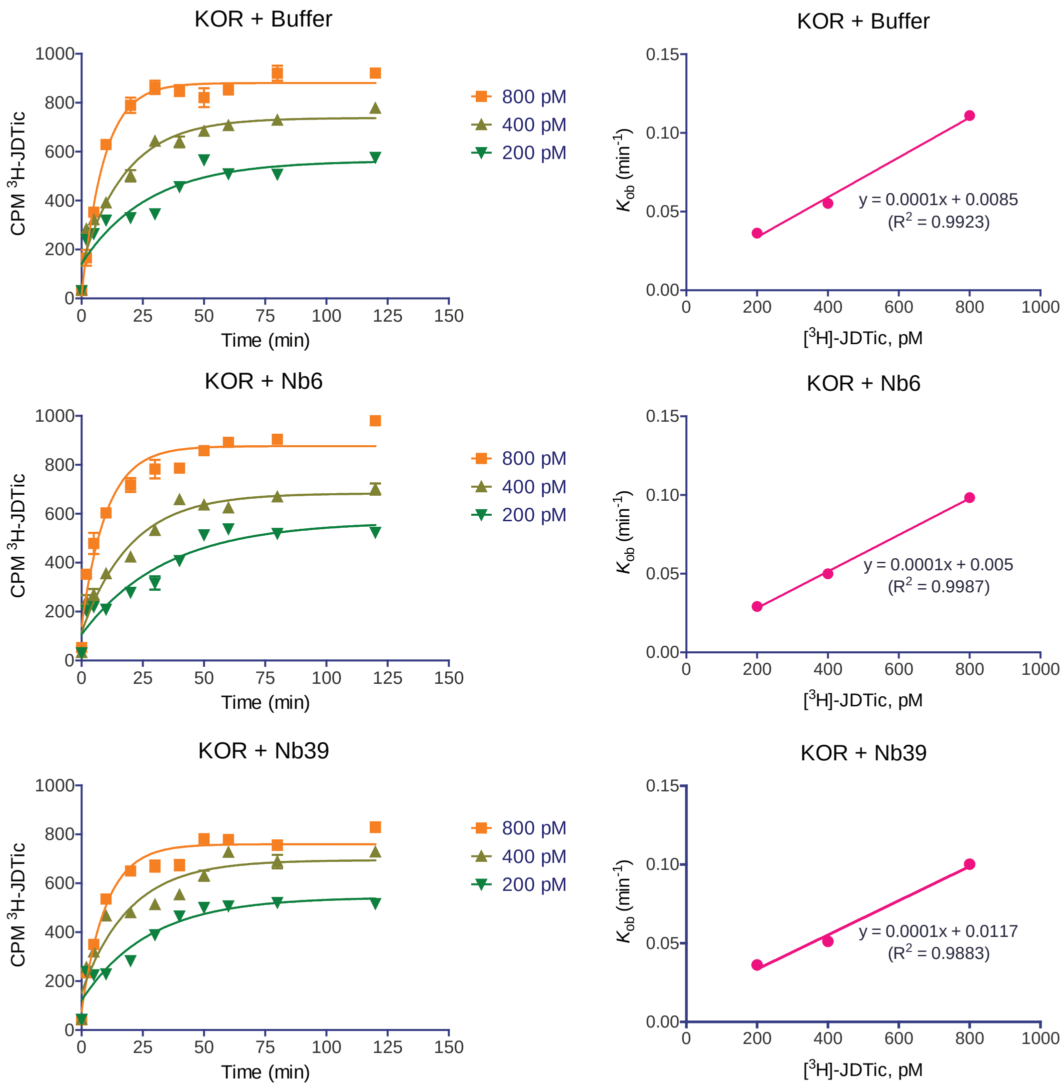

Affinity Values and Kinetically Derived Parameters of JDTic

Membranes	k_{on} ($M^{-1}\,min^{-1}$)	k_{off} (min^{-1})	K_d, nM
KOR + Buffer	1.39×10^8	0.0085	0.061
KOR + Nb6	1.20×10^8	0.0050	0.041
KOR + Nb39	1.22×10^8	0.0117	0.095

Supplementary Figure 2. An alternative assay to measure JDTic kinetics in the presence of Nb6 or Nb39. The k_{on} was determined by incubation of KOR cell membranes with the indicated concentrations of [^{3}H]-JDTic for various time periods. Each point is the mean ± SEM of three experiments. Data were fitted using a one-phase exponential association function to yield an observed on-rate (k_{ob}) (left panel). k_{ob} plotted against the [^{3}H]-JDTic concentration, employed for indirect determination of k_{off} (y-intercept = 0), K_{on} = (K_{ob} - k_{off})/[ligand] (right panel). Source data are provided as a Source Data file.

a

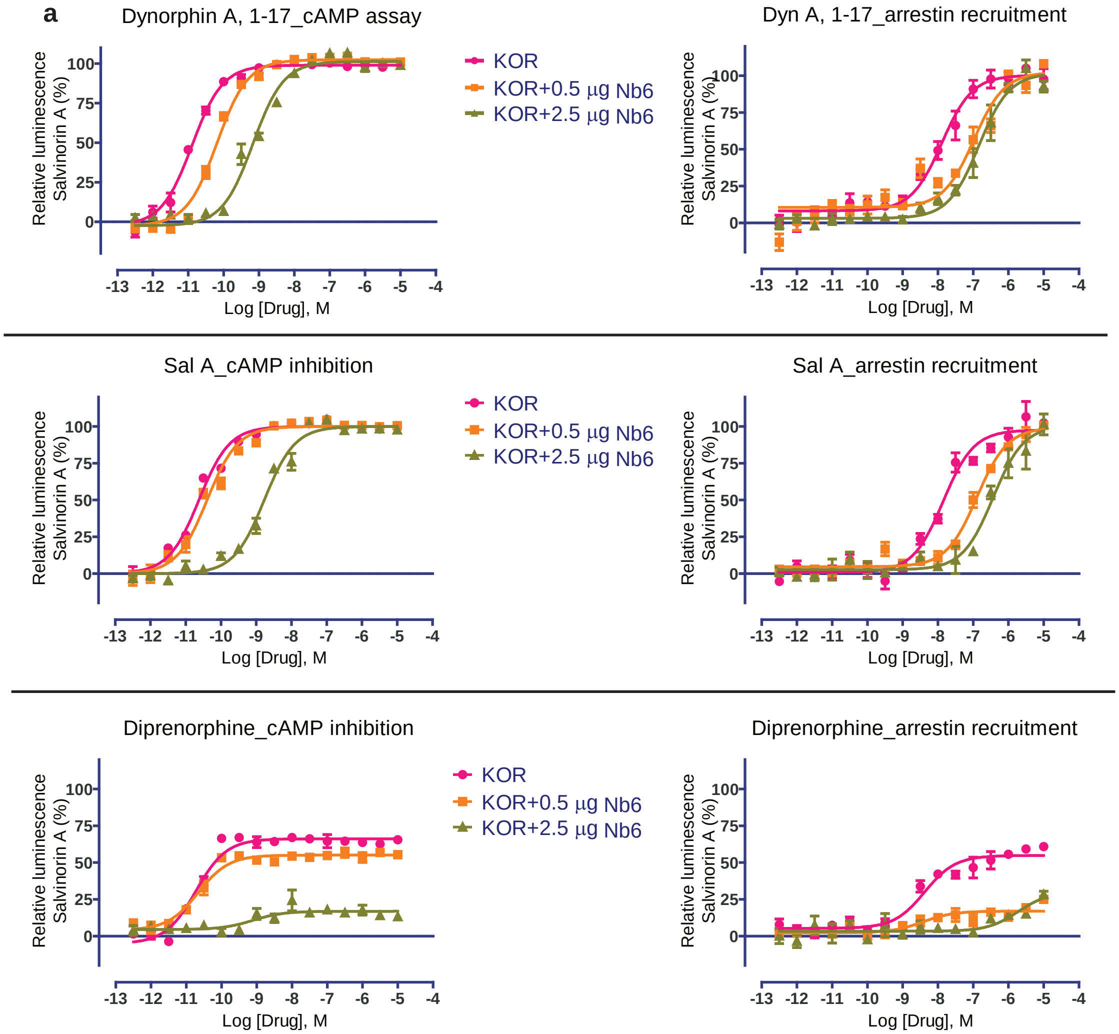

b

KOR ligands		cAMP inhibition assay		Tango arrestin recruitment	
		EC_{50}, nM	E_{max} %	EC_{50}, nM	E_{max} %
Dyn A, 1-17	apo	0.013 ± 0.001	99 ± 1	12.8 ± 1.8	100 ± 3
	+ 0.5 μg Nb6	0.066 ± 0.004	102 ± 1	105 ± 20	102 ± 5
	+ 2.5 μg Nb6	0.68 ± 0.04	101 ± 2	145 ± 19	101 ± 3
Sal A	apo	0.022 ± 0.002	100 ± 1	14.2 ± 2.0	97 ± 3
	+ 0.5 μg Nb6	0.045 ± 0.008	100 ± 1	127 ± 12	99 ± 2
	+ 2.5 μg Nb6	1.69 ± 0.18	100 ± 2	367 ± 55	100 ± 5
Diprenorphine	apo	0.018 ± 0.002	66 ± 1	4.22 ± 0.09	55 ± 2
	+ 0.5 μg Nb6	0.021 ± 0.003	54 ± 1	3.68 ± 1.54	17 ± 1
	+ 2.5 μg Nb6	0.77 ± 0.40	17 ± 2	1910 ± 255	30 ± 7

Supplementary Figure 3. Differential effects of Nb6 on the functional activity of KOR ligands. cAMP inhibition assay and Tango-arrestin recruitment assay were used to measure the effects on G protein activation and arrestin translocation, respectively. Nb6 plasmid DNA at the indicated concentration (0, 0.5, 2.5 μg) was co-transfected with KOR plasmids in HEK 293T cells. **(a)** Nb6 decreases the potency or efficacy of KOR agonist Dynorphin A (1-17), Sal A, and partial agonist diprenorphine. **(b)** Table representing the potency and efficacy value. (N=3, three experiments each done in triplicate). Source data are provided as a Source Data file.

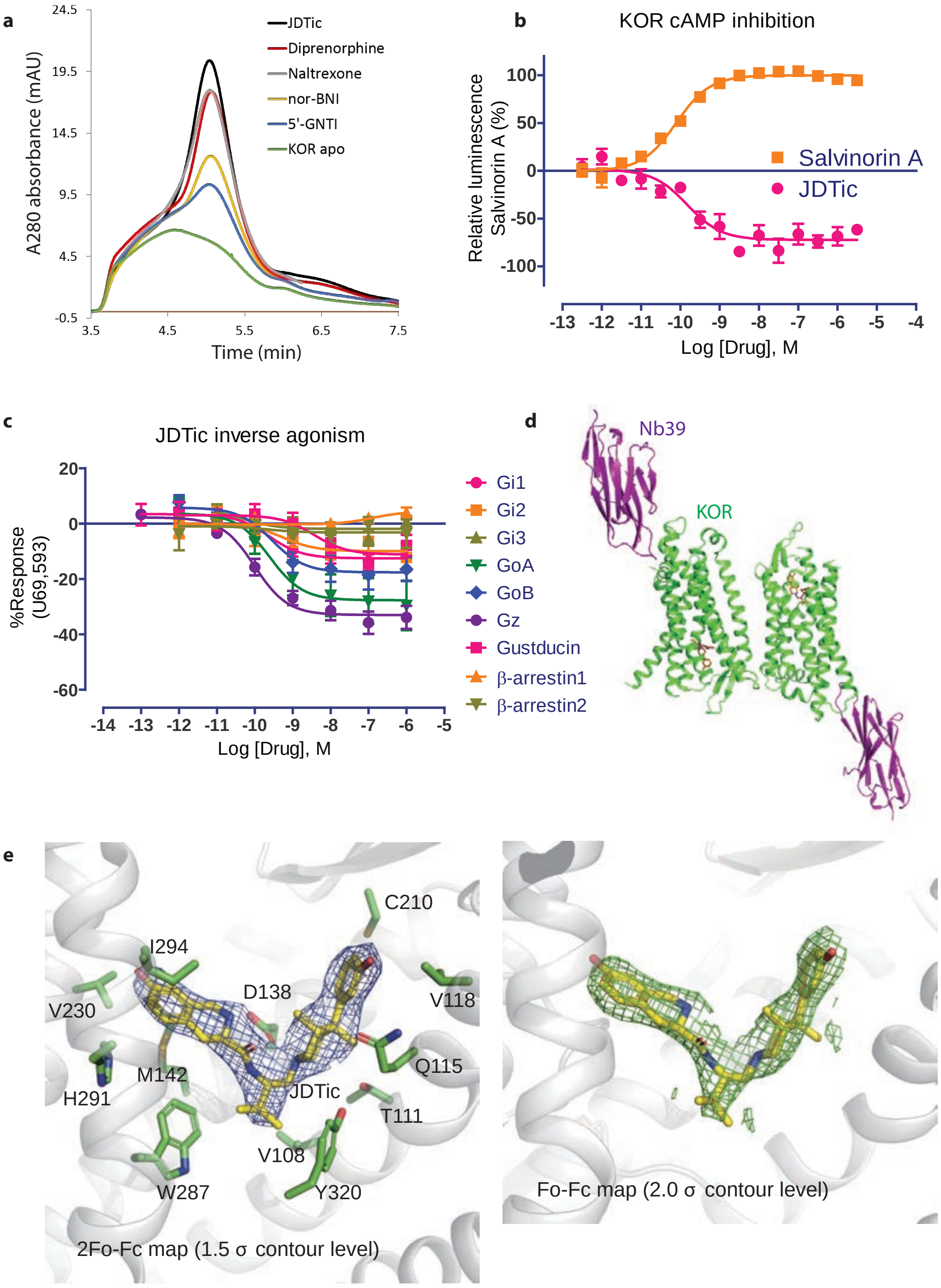

Supplementary Figure 4. The binding pose of the inverse agonist JDTic. (a) JDTic increases the stability of KOR compared with the apo state. KOR proteins were co-purified with different ligands and protein quality was analyzed by size-exclusion chromatography. **(b)** cAMP inhibition assay indicates that JDTic is an inverse agonist at KOR. Data were normalized to the agonist Sal A which could activate Gi/o-coupled KOR receptors, leading to decreased cAMP level. (N=3, three experiments each done in triplicate). **(c)** BRET-based GPCR transducerome screening confirms the inverse agonism of JDTic. The KOR and individual Gi/o family protein (e.g., Gi, Go, Gz) or β-arrestins (β-arrestin1 or 2) were co-transfected into HEK 293T cells. Data were normalized to the agonist U69,593. (N=3, three experiments each done in triplicate). **(d)** The KOR-JDTic-Nb6 structure displays an anti-parallel receptor dimer in the asymmetric unit. **(e)** Electron density of JDTic in the binding pocket with 2mFo-DFc density contoured at 1.5 σ (left) or mFo-DFc omit density map contoured at 2 σ (right). Source data are provided as a Source Data file.

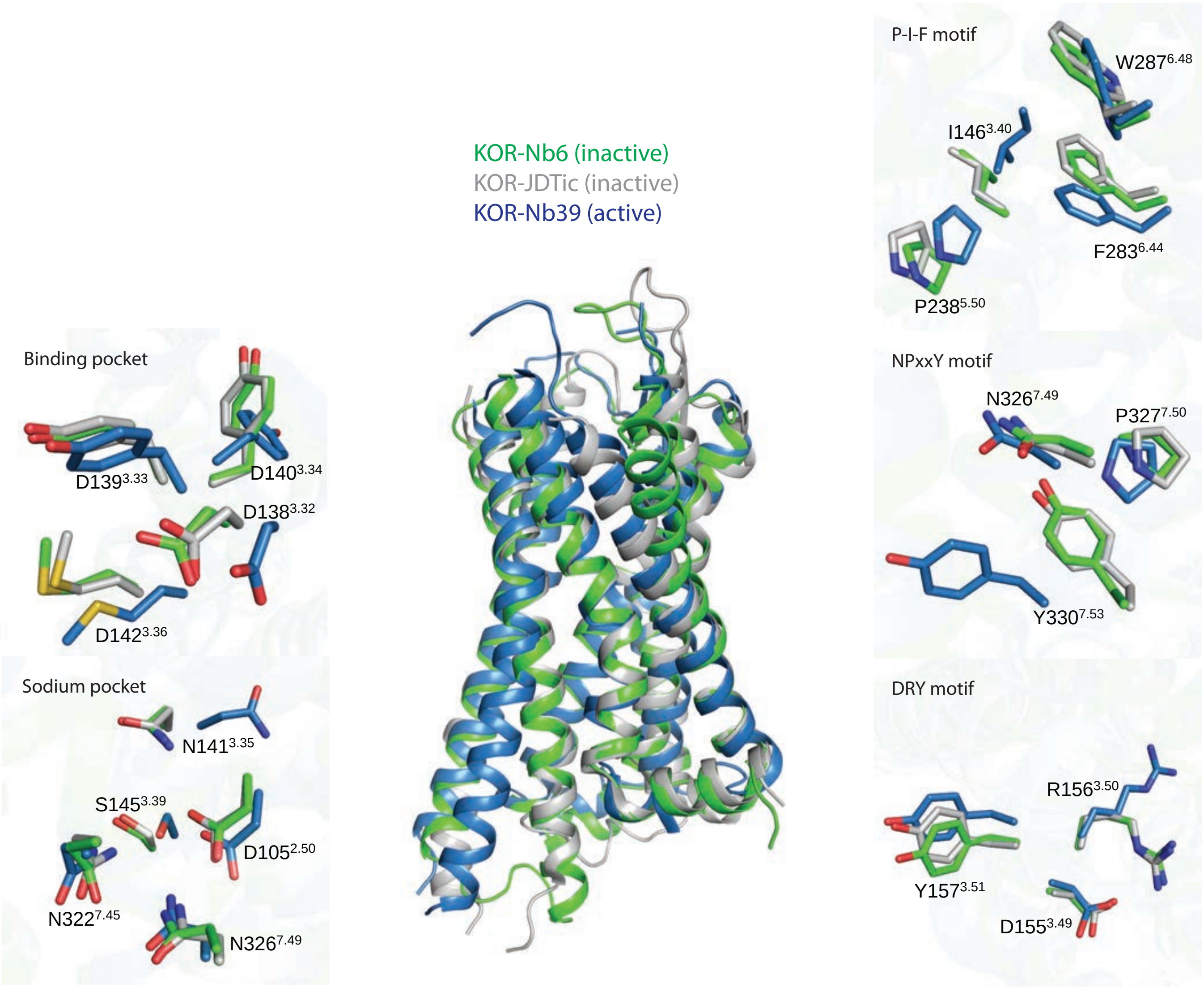

Supplementary Figure 5. Comparison of conserved motifs between KOR-Nb6, KOR-JDTic and KOR-Nb39. Conformational changes between active KOR (blue) and inactive KOR-Nb6 (green) and KOR-JDTic (gray) are highlighted for JDTic binding pocket, sodium binding pocket, P-I-F motif, NPxxY motif, and DRY motif.

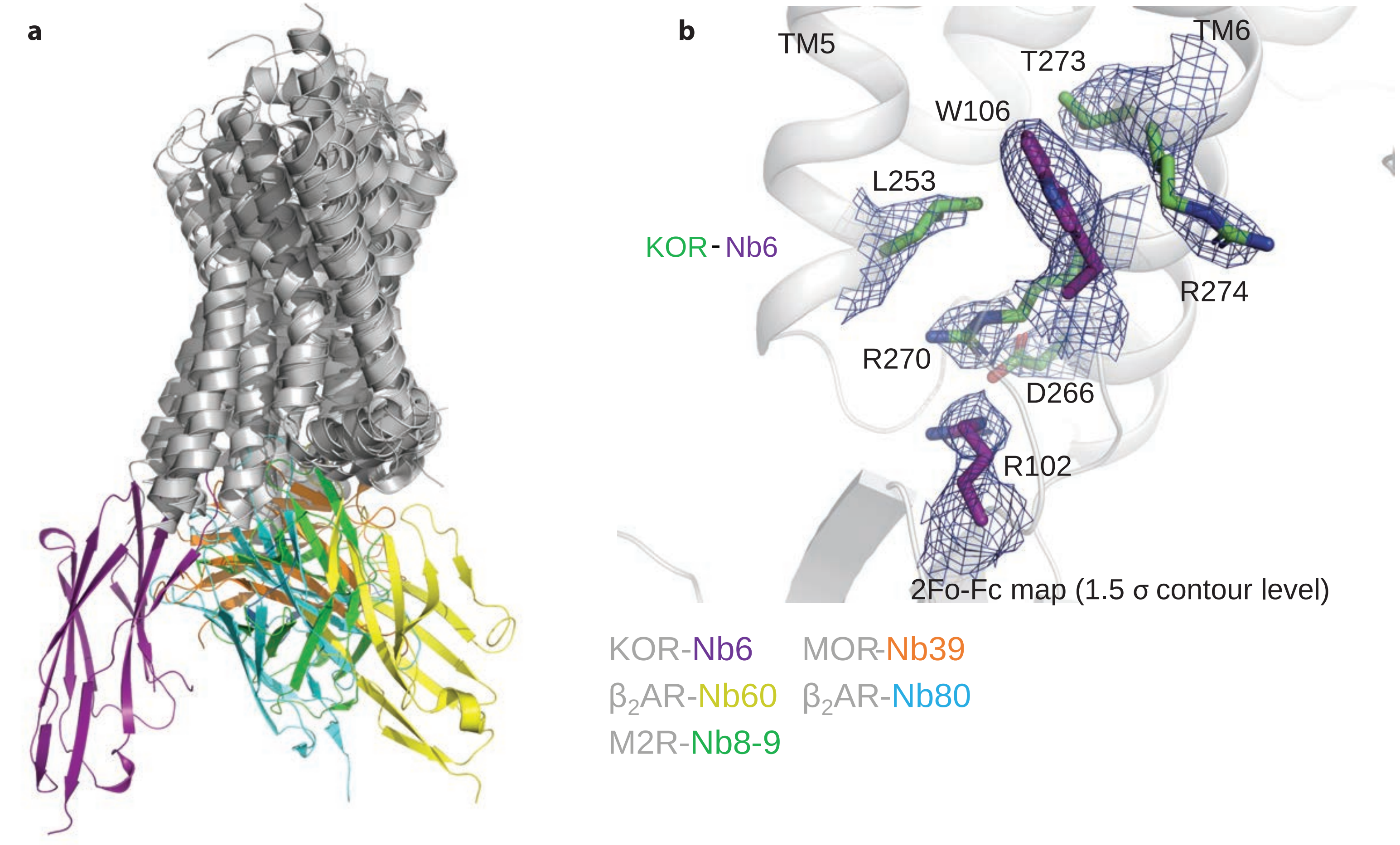

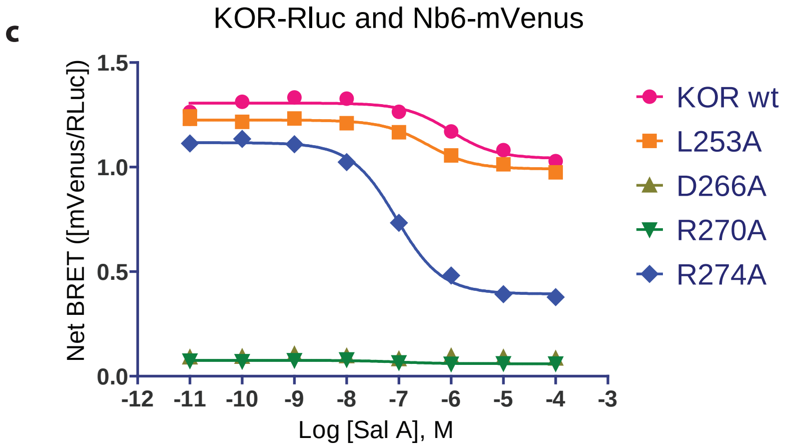

Mutations	EC_{50}, nM
KOR wt	963 ±154
L253A	350 ± 42
D266A	Not measurable
R270A	Not measurable
R274A	89.3 ± 4.5

Supplementary Figure 6. The unique binding pose of Nb6 with KOR. (a) Comparison of nanobody binding pose between KOR-Nb6 (green), KOR-Nb39 (orange, PDB ID 6B73), MOR-Nb39 (orange, PDB ID 5C1M), β_2AR-Nb60 (yellow, PDB ID 5JQH), β_2AR-Nb80 (blue, PDB ID 3P0G) and M2R-Nb8-9 (green, PDB ID 4MQT). **(b)** Electron density map of key residues in the KOR-Nb6 binding interface. Map with 2mFo-DFc density contoured at 1.5 σ. **(c)** BRET assays showing key residues responsible for KOR/Nb6 interactions. EC_{50} values are summarized in the table. (N=3, three experiments each done in triplicate). Source data are provided as a Source Data file.

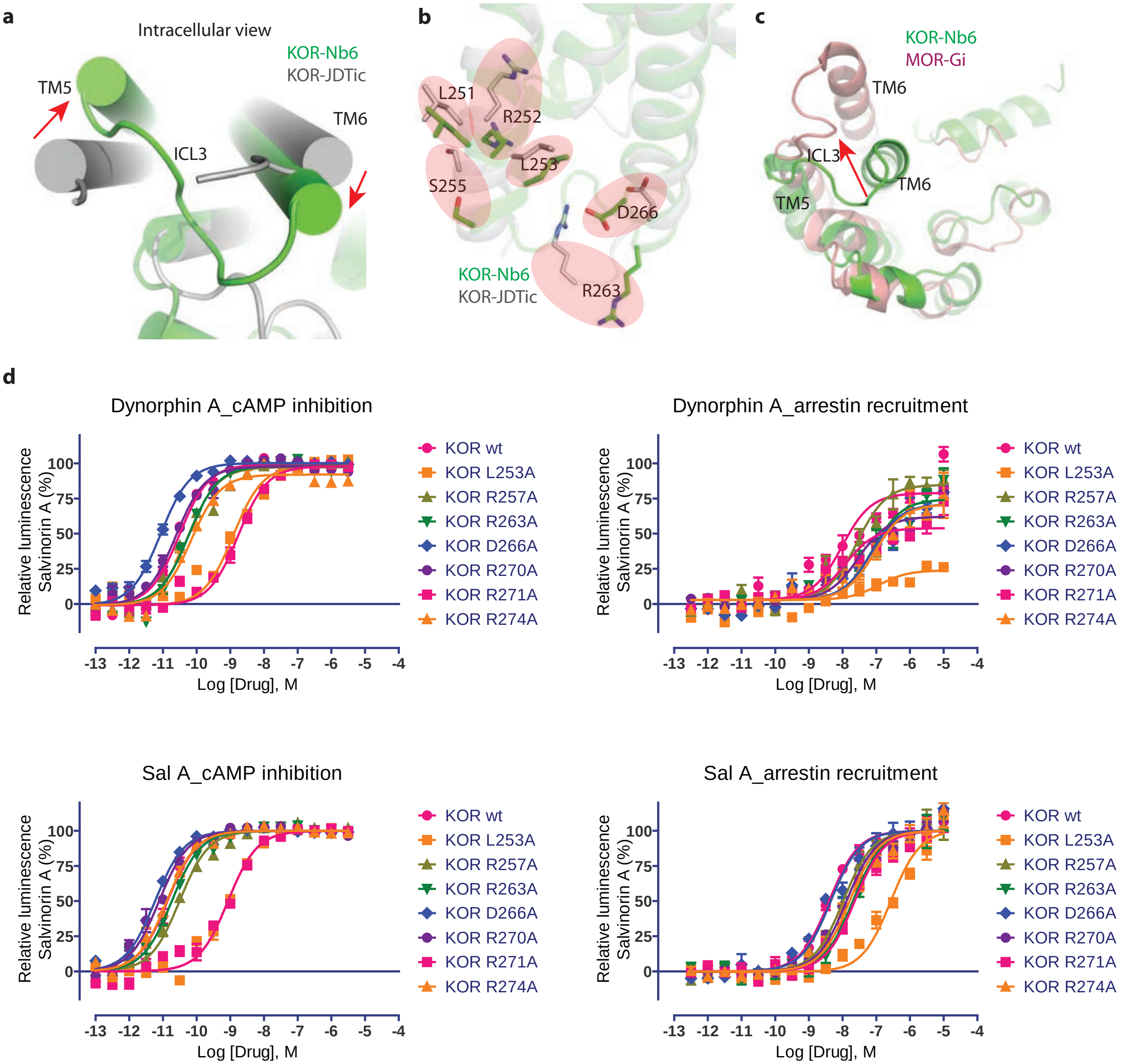

Supplementary Figure 7. Overall comparison between KOR-Nb6, KOR-JDTic and MOR-Gi complex structures. (a) Distortion of intracellular end of TM5 and TM6 upon Nb6 binding. **(b)** Displacement of side chains of residues in the KOR-Nb6 interface. Only residues that have clear electron density in both KOR-Nb6 and KOR-JDTic structures to clarify their conformation are shown. **(c)** A large outward movement of ICL3 during receptor activation. **(d)** Effects of mutations of residues in the KOR-Nb6 interface. EC_{50} and E_{max} values are quantified in Supplementary Table 3. Data were normalized to the agonist Sal A. (N=3, three experiments each done in triplicate).

a

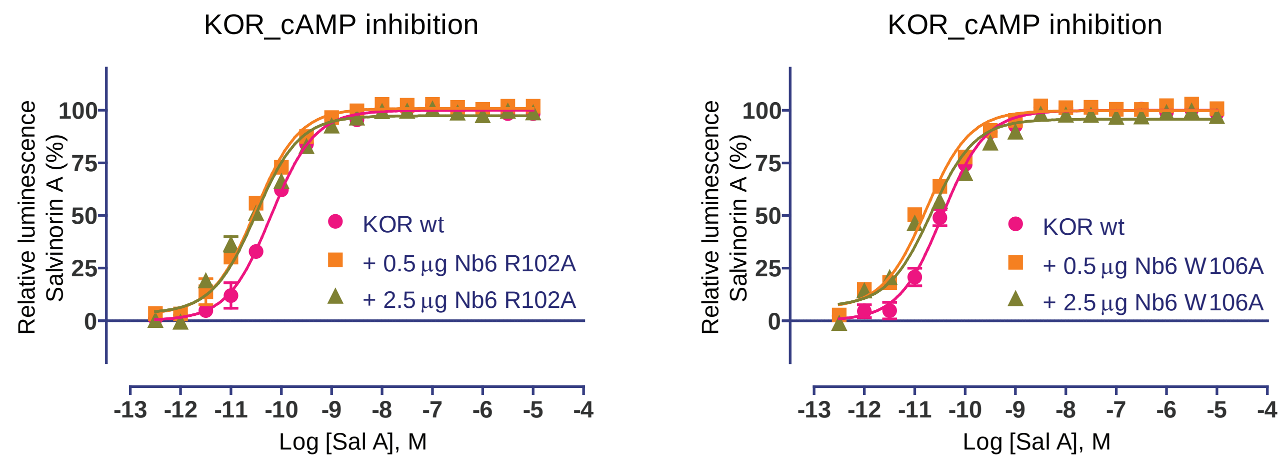

b

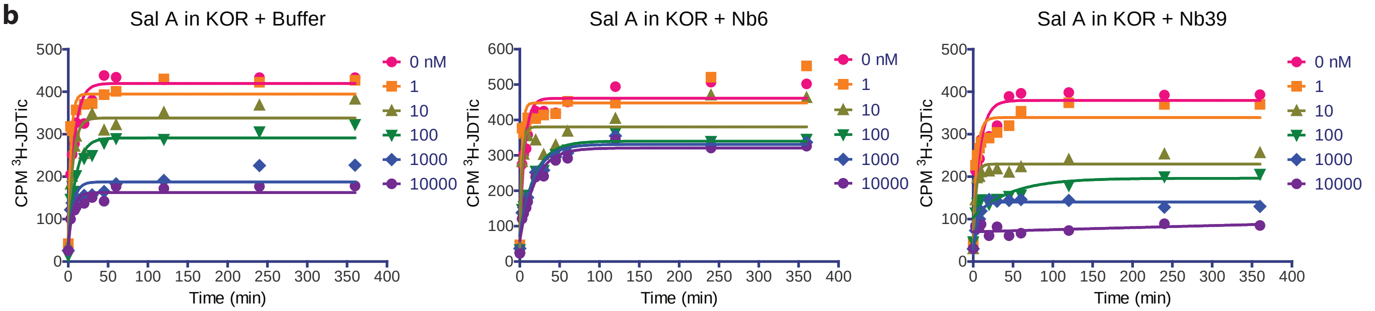

Sal A Dissociation and Association Rates at Wild-Type, Nb6- and Nb39-stabilized KOR.			
Receptor	Half life Time, min (k_{off} ± SEM) min^{-1}	k_{on} ± SEM, M^{-1} min^{-1}	K_d, nM (pKd ± SEM)
KOR wt	15 (0.065 ± 0.005)	$5.96 \times 10^6 \pm 4.36 \times 10^6$	10.9 (7.93 ± 0.42)
KOR + Nb6	6 (0.16 ± 0.04)	$6.83 \times 10^5 \pm 6.45 \times 10^4$	234 (6.63 ± 0.23)
KOR + Nb39	100 (0.01 ± 0.003)	$9.88 \times 10^6 \pm 4.78 \times 10^5$	1.01 (9.00 ± 0.56)

Supplementary Figure 8. Key residues responsible for Nb6's allosteric activity and binding kinetics of Sal A in the presence of Nb 6 or Nb39. (a) Mutation of R102 and W106 in Nb6 abolishes its inhibition activity in KOR-mediated cAMP inhibition. Nb6 plasmid DNA at the indicated concentration (0, 0.5, 2.5 µg) was co-transfected with KOR plasmids in HEK 293T cells. Data were normalized to the agonist Sal A. (N=3, three experiments each done in triplicate). **(b)** Association and dissociation rate of Sal A in the presence of Nb6 or Nb39. Data were acquired by association and dissociation kinetic experiments conducted in parallel at room temperature using [^{3}H]-JDTic (concentration range 0.6–1.5 nM). Estimates of k_{off}, k_{on}, and K_d were obtained from three independent experiments performed in duplicate. The residence time was calculated as $1/k_{off}$. (N=3, three experiments each done in duplicate). Source data are provided as a Source Data file.

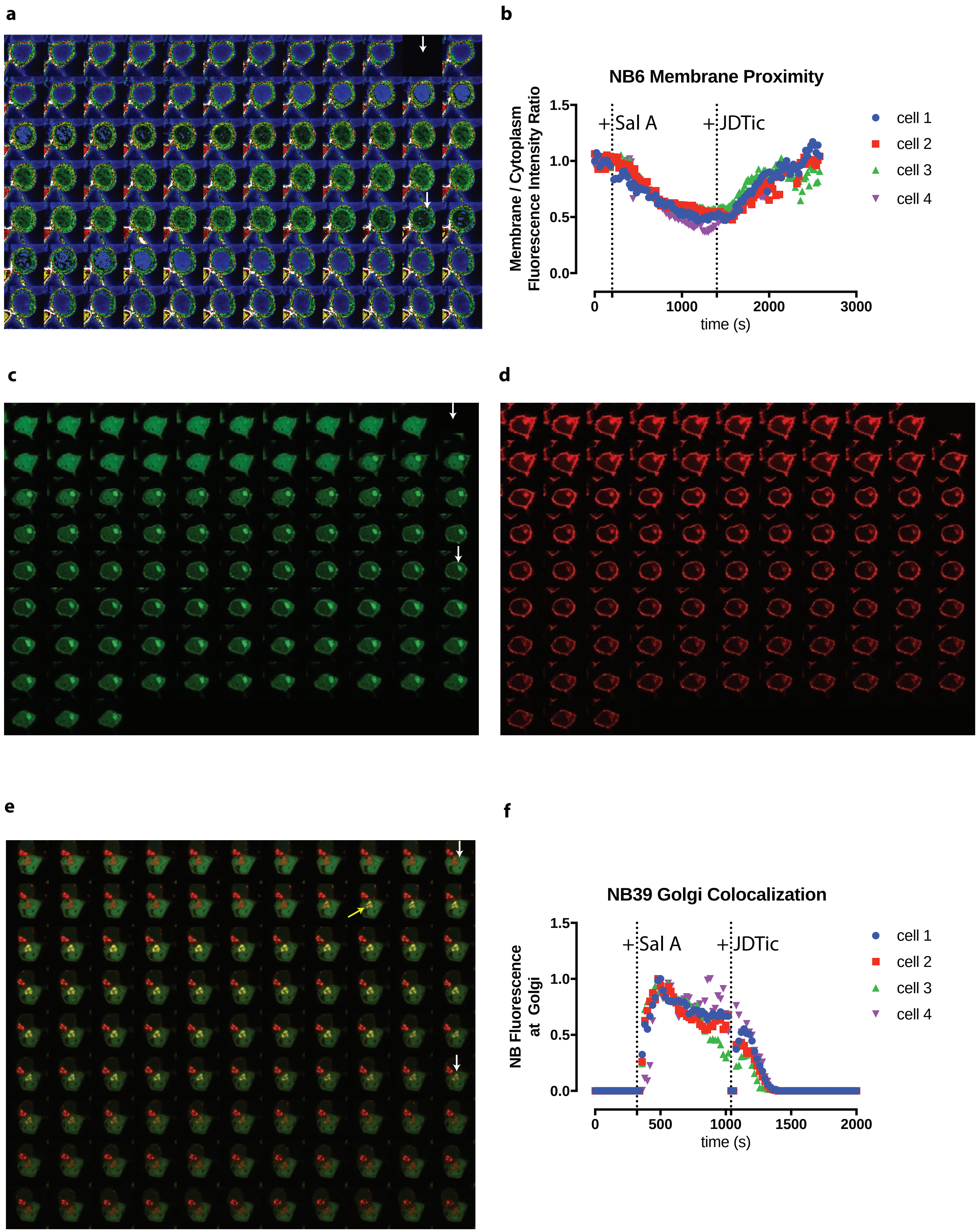
a
b
NB6 Membrane Proximity
Membrane / Cytoplasm Fluorescence Intensity Ratio
+Sal A
+JDTic
cell 1
cell 2
cell 3
cell 4
1.5
1.0
0.5
0.0
0
1000
2000
3000
time (s)
c
d
e
f
NB39 Golgi Colocalization
NB Fluorescence at Golgi
+Sal A
+JDTic
cell 1
cell 2
cell 3
cell 4
1.5
1.0
0.5
0.0
0
500
1000
1500
2000
time (s)

Supplementary Figure 9. Confocal live-cell imaging shows the dynamic translocation of nanobodies. (a) Confocal images of a time series of HEK 293T cells expressing KOR-mScarlet and Nb6-mVenus. Only the intensity of Nb6-mVenus (green) in the cytoplasm was shown here. Blue fluorescence is shown as background.10 μM Sal A and JDTic were added at time points indicated by top and bottom white arrow, respectively. **(b)** Quantification and kinetics of Nb6-mVenus intensity on the cell membrane. $T_{1/2}$ = 484 ± 17 s for Sal A-induced dissociation. $T_{1/2}$ = 512 ± 58 s for JDTic-induced re-association. (**c** and **d**) Confocal images of a time series of HEK 293T cells expressing Nb6-mVenus **(c)** and KOR-mScarlet **(d)**. 10 μM Sal A and JDTic were added at time points indicated by top and bottom white arrow, respectively. **(e)** Rapid activation of Golgi-localized KOR by agonist Sal A. Nb39-mVenus and GalT-RFP were co-transfected with non-labeled KOR in HEK 293T cells. The yellow arrow indicates the co-localization of the Nb39 and Golgi apparatus. 10 μM Sal A and JDTic were added at time points indicated by top and bottom white arrow, respectively. Nb39-mVenus and GalT-RFP show co-localization after Sal A stimulation. **(f)** Quantification and kinetics of Nb39-mVenus intensity on the Golgi apparatus. $T_{1/2}$ = 75 ± 5 s for Sal A-induced association. $T_{1/2}$ = 661 ± 28 s for JDTic-induced dissociation. (N=3, three experiments each done in triplicate).

a

	5.65	6.24	6.27	6.31	6.32	6.35	ICL3
KOR	L253	R263	D266	R270	R271	R274	R257
MOR	L261	K271	D274	R278	R279	R282	R265
DOR	L240	K250	D253	R257	R258	R261	R244
NOP	L242	R252	D255	R259	R260	R263	R246

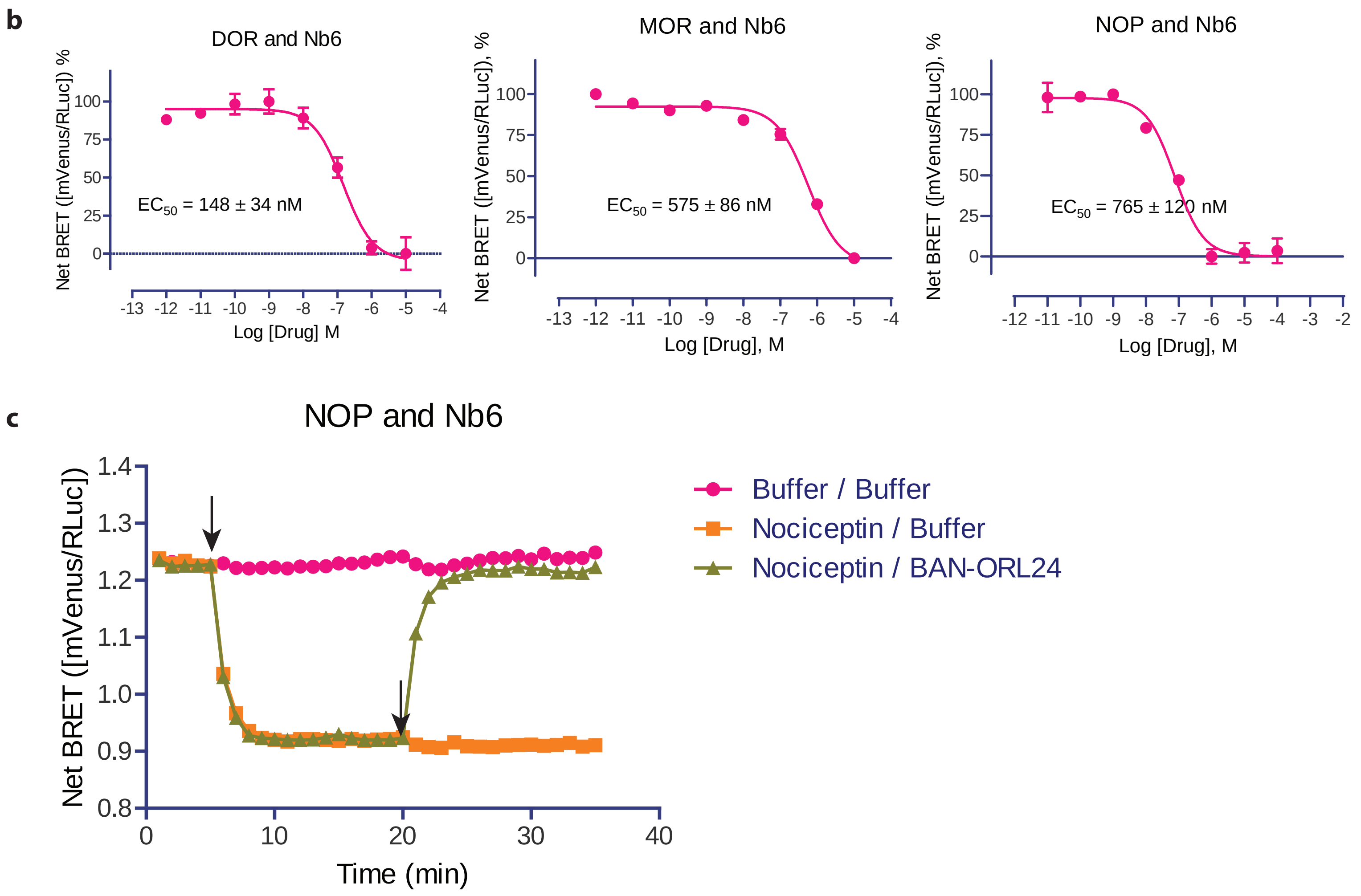

Supplementary Figure 10. Nb6 can bind to all four opioid receptors. (a) Residues on the KOR-Nb6 interface are highly conserved in MOR, DOR, and NOP receptors. **(b)** Screening of Nb6 over other opioid receptors in BRET assay confirms that Nb6 also binds to other opioid receptors. (N=3, three experiments each done in triplicate). **(c)** Nb6 can robustly report activation and inactivation of nociception receptors. The first and second arrows indicate the time when the agonist nociception and antagonist BAN-ORL24 were added, respectively. (N=3, three experiments each done in triplicate). Source data are provided as a Source Data file.

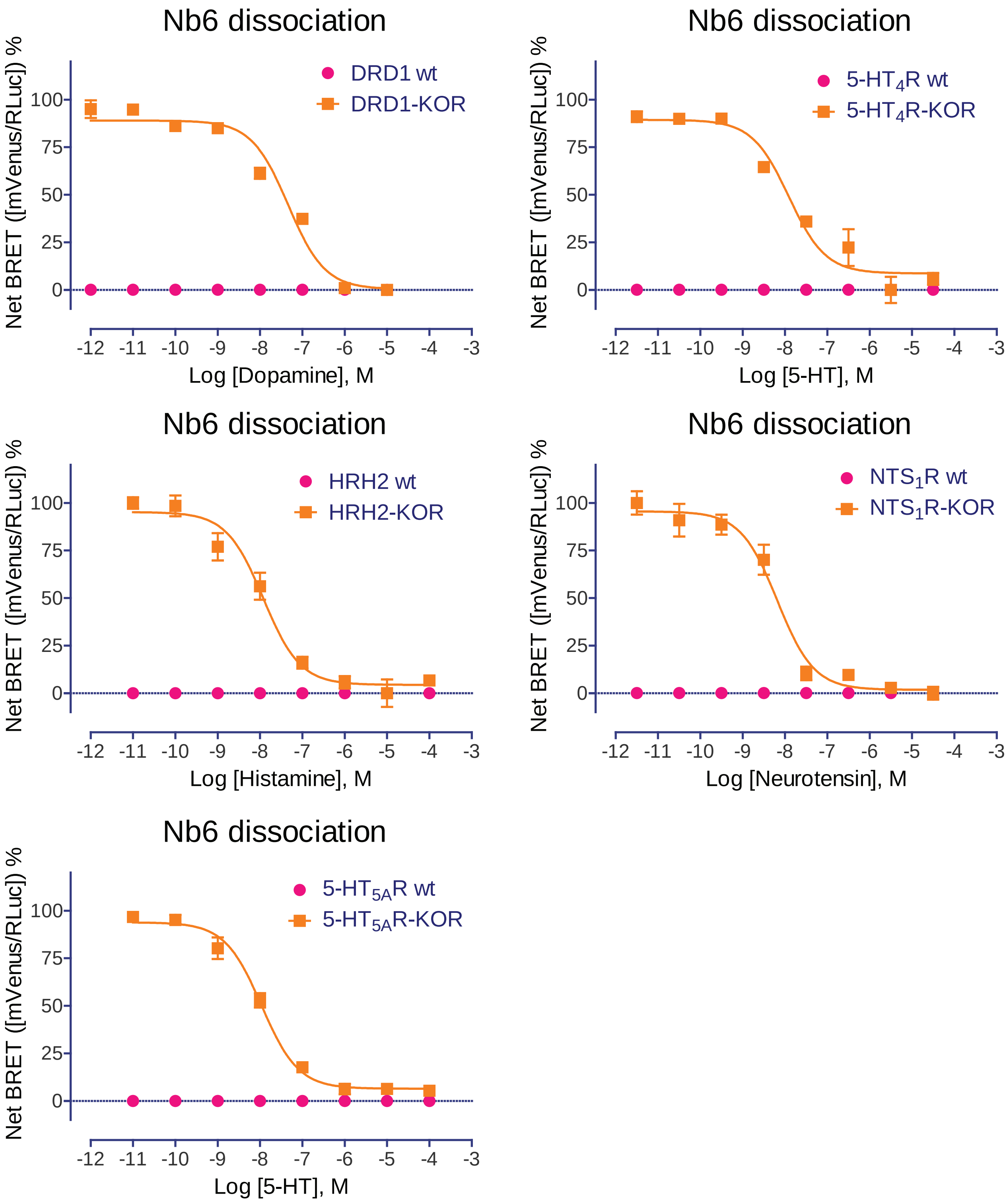

Supplementary Figure 11. The GPCR-KOR chimera approach allows Nb6 to bind to other G protein-coupled receptors. Gs-coupled [D1 dopamine (DRD1), human serotonin 5-HT_4 (5-HT_4R), Histamine 2 (HRH2)], Gi-coupled [neurotensin-1 (NTSR-1), human serotonin 5A (5-HT_{5A}R)] receptors have been engineered to via the chimera approach. These chimera receptors were then tested in BRET assay in the presence of their respective endogenous ligands. The EC50 values were summarized in Supplementary Table 4. (N=3, three experiments each done in triplicate). Source data are provided as a Source Data file.

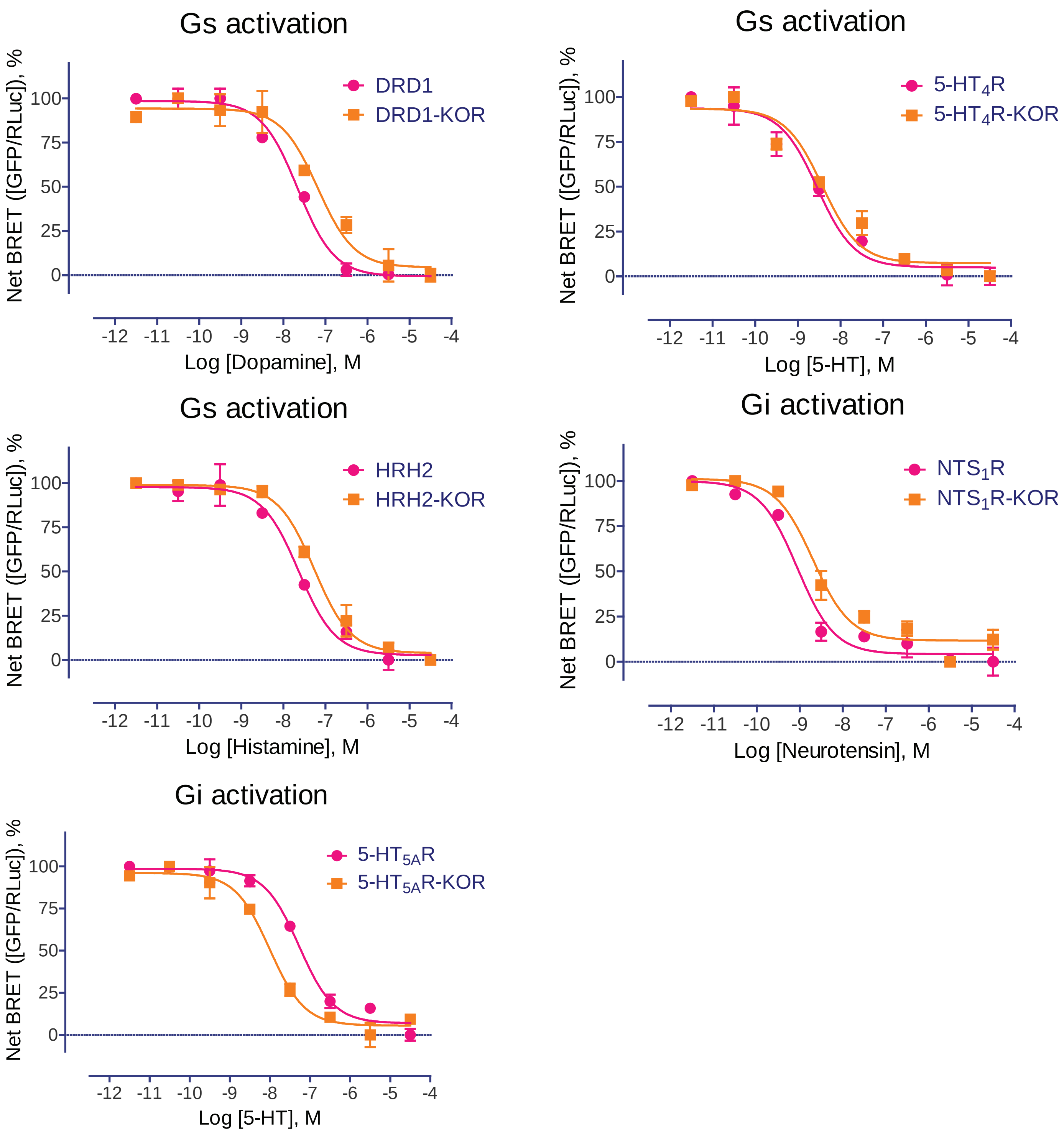

Supplementary Figure 12. Comparison of G protein coupling between wild type and KOR chimeric GPCRs. Wild type and chimeric receptors were tested in parallel with their canonical G proteins in BRET-based assays. The EC50 values were summarized in Supplementary Table 5. (N=3, three experiments each done in triplicate). Source data are provided as a Source Data file.

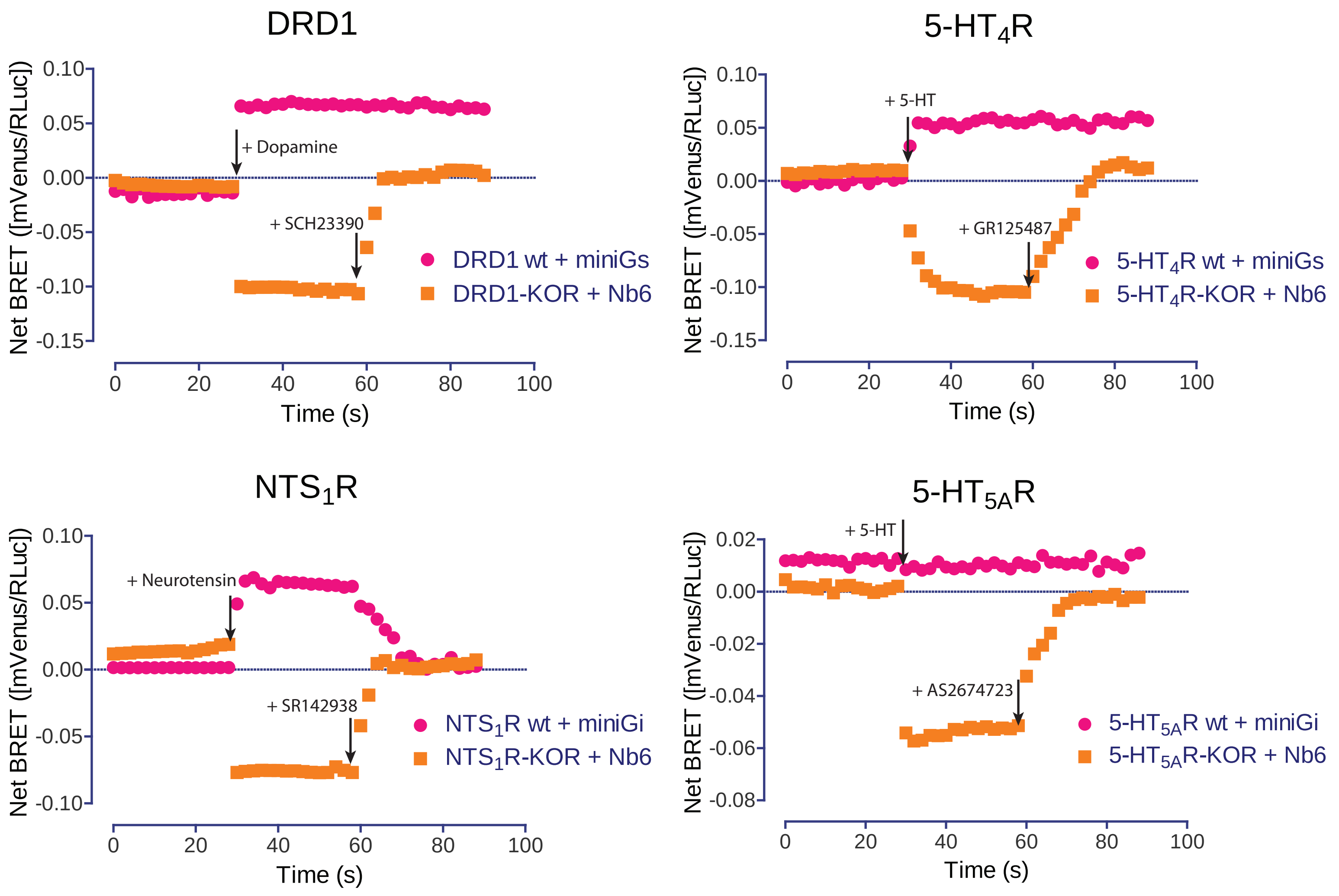

Supplementary Figure 13. Comparison of kinetics monitored by Nb6 and miniG proteins using BRET-based assays. The first and second arrows are the time when the agonist and the antagonist were added, respectively. For different receptors tested, the agonist/antagonist were Dopamine/SCH23390 for DRD1, 5-HT/GR125487 for $5HT_4R$, Neurotensin/SR142948 for NTS_1R, and 5-HT/AS2674723[58] for $5\text{-}HT_{5A}R$. (N=3, three experiments each done in triplicate). Source data are provided as a Source Data file.

Supplementary Table 1. Data Collection and Refinement Statistics.

**Structure**	**BRIL-KOP-JDTic-Nb6**	
**Data Collection**	APS, GMCA/CAT 23ID-B/D, 1.033 Å, 10-µm	
Crystals	31	
Resolution (Å)	30.00-3.30 (3.43-3.30)	
Space group	$P2_1 2 2_1$	
Complexes/ASU	2	
Unit cell dimensions *a, b, c* (Å)	102.39, 108.19, 155.1	
α, β, γ (°)	90, 90, 90	
No. total reflections	174,722	
No. unique reflections	26,340 (1,998)	
Multiplicity	6.6 (6.6)	
Completeness (%)	99.1 (99.2)	
Mean I/σ(I)	9.9 (1.1)	
R_{merge} (%)	18.2 (111.4)	
$CC_{1/2}$ (%)	87.4 (48.0)	
**Refinement Statistics**		
Resolution used in refinement (Å)	30.00-3.30 (3.43-3.30)	
No. reflections used in refinement	26,279 (2,518)	
No. reflections used for R-free	1,282 (114)	
R-work (%)	23.9 (31.8)	
R-free (%)	27.0 (40.4)	
**Number of atoms**	Complex A	Complex B
KOP	2,136	2,115
Nb6	866	862
JDTic	34	34
Lipids		28
***Overall B*-factors (Å^2)**	Complex A	Complex B
KOP	84.9	89.6
Nb6	87.5	88.0
JDTic	76.1	94.1
Lipids		85.7
Model Statistics		
RMSD Bond (Å)	0.010	
RMSD Bond (º)	1.24	
Ramachandran Favored (%)[a]	96.01	
Ramachandran Allowed (%)[a]	3.99	
Ramachandran Outliers (%)[a]	0.0	
Rotamer outliers (%)[a]	0.0	
Molprobity score[a]	1.81	

The highest-resolution shell is shown in parentheses.

[a]As defined in MolProbity.

Supplementary Table 2. Binding affinity (Ki) of KOR ligands in the presence of Nb6 or Nb39. This is a summary of the values in Figure 1. Data represent mean Ki ± SEM and experiments were performed in triplicate.

Ligands	Ki ± SEM, nM		
	KOR	KOR + Nb6	KOR + Nb39
JDTic	0.20 ± 0.03	0.21 ± 0.03	0.17 ± 0.02
Dynorphin A	11.1 ± 0.6	107 ± 16	1.98 ± 0.21
LY2459989	3.40 ± 0.25	0.51 ± 0.03	6.93 ± 0.82

Supplementary Table 3. EC_{50}s and Emax of agonists SalA and Dynorphin A in hKOR wild type and mutants of residues in the KOR-Nb6 interface. Data represent mean EC_{50} (pEC_{50} ± SEM) or Emax ± SEM % and experiments were performed in triplicate.

KOR Mutations	Dynorphin A, 1-17				Sal A			
	cAMP inhibition assay		Tango arrestin recruitment		cAMP inhibition assay		Tango arrestin recruitment	
	EC_{50}, nM (pEC_{50} ± SEM)	E_{max} ± SEM %	EC_{50}, nM (pEC_{50} ± SEM)	E_{max} ± SEM %	EC_{50}, nM (pEC_{50} ± SEM)	E_{max} ± SEM %	EC_{50}, nM (pEC_{50} ± SEM)	E_{max} ± SEM %
KOR wt	0.031 (10.51 ± 0.04)	99 ± 1	6.53 (8.18 ± 0.09)	71 ± 3	0.014 (10.87 ± 0.05)	100 ± 1	4.00 (8.40 ± 0.05)	100 ± 2
L253A	1.22 (8.91 ± 0.05)	98 ± 1	59.7 (7.22 ± 0.37)	21 ± 4	0.85 (9.07 ± 0.04)	99 ± 2	273 (6.56 ± 0.09)	100 ± 4
R257A	0.056 (10.51 ± 0.04)	97 ± 1	23.6 (7.63 ± 0.08)	83 ± 3	0.031 (10.51 ± 0.04)	99 ± 2	10.4 (7.98 ± 0.07)	99 ± 1
R263A	0.058 (10.24 ± 0.04)	98 ± 1	39.2 (7.41 ± 0.11)	64 ± 3	0.018 (10.74 ± 0.04)	100 ± 1	20.3 (7.69 ± 0.08)	100 ± 3
D266A	0.008 (11.07 ± 0.04)	100 ± 1	48.5 (7.31 ± 0.11)	65 ± 3	0.006 (11.23 ± 0.03)	100 ± 1	4.47 (8.35 ± 0.08)	100 ± 3
R270A	0.024 (10.61 ± 0.05)	97 ± 1	14.9 (7.83 ± 0.13)	53 ± 3	0.008 (11.11 ± 0.05)	99 ± 1	13.1 (7.88 ± 0.06)	100 ± 2
R271A	1.71 (8.77 ± 0.04)	98 ± 1	5.81 (8.24 ± 0.14)	49 ± 2	0.86 (9.07 ± 0.06)	100 ± 2	23.0 (7.64 ± 0.08)	100 ± 1
R274A	0.070 (10.16 ± 0.05)	92 ± 1	65.9 (7.18 ± 0.11)	66 ± 4	0.013 (10.88 ± 0.04)	100 ± 1	16.2 (7.79 ± 0.07)	100 ± 3

Supplementary Table 4. EC_{50}s of chimeric receptors and Nb6 dissociation upon the agonist treatment. This is a summary of the values in Figure 5 and Supplementary Figure 11. Data represent mean EC_{50} (pEC_{50}± SEM) or Emax %±SEM and experiments were performed in triplicate.

	Receptors	EC_{50}, nM (pEC_{50} ± SEM)
Gαs family	DRD1-KOR	46.09 (7.34 ± 0.21)
	5-HT_4R-KOR	12.31 (7.91 ± 0.15)
	HRH2-KOR	11.97 (7.92 ± 0.12)
Gαi family	NTS_1R-KOR	6.58 (8.18 ± 0.12)
	5-HT_{5A}R-KOR	10.86 (7.96 ± 0.07)
Gαq family	5-HT_{2A}R-KOR	98.97 (7.00 ± 0.08)
	ET_A-KOR	75.31 (7.12 ± 0.06)

Supplementary Table 5. EC_{50}s of chimeric receptors compared with wild type in G protein coupling. This is a summary of the values in Figure 5 and Supplementary Figure 12. Data represent mean EC_{50} (pEC_{50}± SEM) or Emax %±SEM and experiments were performed in triplicate.

G protein activation	Receptors	EC_{50}, nM (pEC_{50} ± SEM)
Gαs	DRD1 wt	22.03 (7.66 ± 0.08)
	DRD1-KOR	65.70 (7.18 ± 0.15)
	5-HT_4R wt	2.85 (8.55 ± 0.13)
	5-HT_4R-KOR	3.76 (8.43 ± 0.15)
	HRH2 wt	23.16 (7.64 ± 0.10)
	HRH2-KOR	54.08 (7.27 ± 0.07)
Gαi	NTS_1R wt	0.87 (9.06 ± 0.12)
	NTS_1R-KOR	2.03 (8.69 ± 0.13)
	5-HT_{5A}R wt	53.21 (7.27 ± 0.09)
	5-HT_{5A}R-KOR	9.9 (8.00 ± 0.10)
Gαq	5-HT_{2A}R wt	6.70 (8.17 ± 0.08)
	5-HT_{2A}R-KOR	7.40 (8.13 ± 0.14)
	ET_A wt	7.71 (8.11 ± 0.10)
	ET_A-KOR	5.54 (8.26 ± 0.16)

HA secretory sequence

Myc sequence

GPCR sequence

KOR insert

SRGG linker

Renilla Luciferase (Rluc8)

1. Human 5-HT2A

Protein sequence
MKTIIALSYIFCLVFAEQKLISEEDLAMDILCEENTSLSSTTNSLMQLNDDTRLYSNDFNS
GEANTSDAFNWTVDSENRTNLSCEGCLSPSCLSLLHLQEKNWSALLTAVVIILTIAGNIL
VIMAVSLEKKLQNATNYFLMSLAIADMLLGFLVMPVSMLTILYGYRWPLPSKLCAVWIYL
DVLFSTASIMHLCAISLDRYVAIQNPIHHSRFNSRTKAFLKIIAVWTISVGISMPIPVFGLQ
DDSKVFKEGSCLLADDNFVLIGSFVSFFIPLTIMVITYFLTIKSLQKVRLLSGSREKDRNL
RRITRLVLVVVAVFVVMWCPFFITNIMAVICKESCNEDVIGALLNVFVWIGYLSSAVNPL
VYTLFNKTYRSAFSRYIQCQYKENKKPLQLILVNTIPALAYKSSQLQMGQKKNSKQDAK
TTDNDCSMVALGKQHSEEASKDNSDGVNEKVSCVSRGGMASKVYDPEQRKRMITGP
QWWARCKQMNVLDSFINYYDSEKHAENAVIFLHGNATSSYLWRHVVPHIEPVARCIIP
DLIGMGKSGKSGNGSYRLLDHYKYLTAWFELLNLPKKIIFVGHDWGAALAFHYAYEHQ
DRIKAIVHMESVVDVIESWDEWPDIEEDIALIKSEEGEKMVLENNFFVETVLPSKIMRKL
EPEEFAAYLEPFKEKGEVRRPTLSWPREIPLVKGGKPDVVQIVRNYNAYLRASDDLPK
LFIESDPGFFSNAIVEGAKKFPNTEFVKVKGLHFLQEDAPDEMGKYIKSFVERVLKNEQ*

DNA sequence
ATGAAGACGATCATCGCCCTGAGCTACATCTTCTGCCTGGTATTCGCCGAACAAAA
GCTCATTTCCGAAGAGGATCTTGCCATGGATATCTTGTGCGAGGAGAATACCTCAC
TGTCCAGCACAACTAACTCTCTCATGCAGCTCAATGATGATACCCGACTTTACTCC
AACGACTTCAACAGTGGTGAGGCTAATACATCAGACGCCTTCAATTGGACAGTAGA
CAGTGAAAACCGGACCAATCTGTCCTGTGAGGGATGCCTGTCCCCAAGTTGTTTGT
CTCTCCTGCATTTGCAGGAAAAGAACTGGTCTGCGCTGCTCACCGCCGTGGTTATC
ATACTCACTATAGCTGGCAATATTCTCGTAATCATGGCCGTGTCCCTGGAAAAAAA
GCTGCAGAACGCCACGAACTACTTCCTGATGTCTCTGGCGATTGCGGATATGCTG
CTTGGGTTTCTGGTGATGCCTGTATCCATGTTGACTATACTGTACGGGTACCGCTG
GCCACTCCCATCCAAGTTGTGTGCCGTCTGGATCTATCTGGACGTTCTCTTTAGTA
CCGCAAGCATAATGCACCTTTGTGCTATTAGCCTGGACCGCTACGTCGCTATTCAA
AACCCCATCCACCACAGCAGATTCAACAGCCGAACTAAAGCCTTTCTGAAAATCAT
CGCGGTCTGGACCATTTCAGTTGGCATCAGCATGCCGATACCTGTATTTGGCCTTC
AGGATGACTCTAAGGTCTTCAAAGAGGGCAGTTGCCTTCTGGCTGATGACAATTTC
GTACTTATAGGAAGCTTCGTGTCCTTCTTCATTCCTCTTACCATTATGGTGATCACT
TACTTCCTGACAATTAAGTCCCTTCAAAAGGTGCGCCTGCTGAGCGGCAGCCGCG

AAAAGGATCGGAATCTGCGCCGAATCACACGCCTCGTGCTGGTTGTAGTCGCTGT
CTTCGTGCTGTGCTGGTTCCCCCTGCATCTGAGCCGCATTCTCAAGAAAACCGTCT
ACAACGAGATGGACAAAAATAGATGCGAATTGCTCTCCTTTTTGCTTTTGATGGACT
ATATTGGGATTAACCTTGCCACCATGAATTCCTGTATCAACCCAATTGCTCTCTACT
TTGTGTCAAAGAAATTCAAGAACTGTTTCCAAAGCTGTCTCTGTTGCTGTTGCTACC
AGTCTAAGAGTCTGATGACCTCAGTGCCCATGAATGGGACAAGTATCCAGTGGAA
GAACCATGACCAGAATAACCACAACACAGACCGCTCATCCCATAAGGATAGCATGA
ACTCTAGAGGGGGGATGGCTTCCAAGGTGTACGACCCCGAGCAACGCAAACGCAT
GATCACTGGGCCTCAGTGGTGGGCTCGCTGCAAGCAAATGAACGTGCTGGACTCC
TTCATCAACTACTATGATTCCGAGAAGCACGCCGAGAACGCCGTGATTTTTCTGCA
TGGTAACGCTACCTCCAGCTACCTGTGGAGGCACGTCGTGCCTCACATCGAGCCC
GTGGCTAGATGCATCATCCCTGATCTGATCGGAATGGGTAAGTCCGGCAAGAGCG
GGAATGGCTCATATCGCCTCCTGGATCACTACAAGTACCTCACCGCTTGGTTCGAG
CTGCTGAACCTTCCAAAGAAAATCATCTTTGTGGGCCACGACTGGGGGGCTGCTC
TGGCCTTTCACTACGCCTACGAGCACCAAGACAGGATCAAGGCCATCGTCCATAT
GGAGAGTGTCGTGGACGTGATCGAGTCCTGGGACGAGTGGCCTGACATCGAGGA
GGATATCGCCCTGATCAAGAGCGAAGAGGGCGAGAAAATGGTGCTTGAGAATAAC
TTCTTCGTCGAGACCGTGCTCCCAAGCAAGATCATGCGGAAACTGGAGCCTGAGG
AGTTCGCTGCCTACCTGGAGCCATTCAAGGAGAAGGGCGAGGTTAGACGGCCTAC
CCTCTCCTGGCCTCGCGAGATCCCTCTCGTTAAGGGAGGCAAGCCCGACGTCGTC
CAGATTGTCCGCAACTACAACGCCTACCTTCGGGCCAGCGACGATCTGCCTAAGC
TGTTCATCGAGTCCGACCCTGGGTTCTTTTCCAACGCTATTGTCGAGGGAGCTAAG
AAGTTCCCTAACACCGAGTTCGTGAAGGTGAAGGGCCTCCACTTCCTCCAGGAGG
ACGCTCCAGATGAAATGGGTAAGTACATCAAGAGCTTCGTGGAGCGCGTGCTGAA
GAACGAGCAGTAA

2. Human ET_A

Protein sequence
MKTIIALSYIFCLVFAEQKLISEEDLAMETLCLRASFWLALVGCVISDNPERYSTNLSNHV
DDFTTFRGTELSFLVTTHQPTNLVLPSNGSMHNYCPQQTKITSAFKYINTVISCTIFIVG
MVGNATLLRIIYQNKCMRNGPNALIASLALGDLIYVVIDLPINVFKLLAGRWPFDHNDFG
VFLCKLFPFLQKSSVGITVLNLCALSVDRYRAVASWSRVQGIGIPLVTAIEIVSIWILSFIL
AIPEAIGFVMVPFEYRGEQHKTCMLNATSKFMEFYQDVKDWWLFGFYFCMPLVCTAV
CYTLMILRLKSVRLLSGSREKDRNLRRITRLVLVVVAVFVLCWFPLHLSRILKKTVYNEM
DKNRCELLSFLLLMDYIGINLATMNSCINPIALYFVSKKFKNCFQSCLCCCCYQSKSLMT
SVPMNGTSIQWKNHDQNNHNTDRSSHKDSMNSRGGMASKVYDPEQRKRMITGPQW
WARCKQMNVLDSFINYYDSEKHAENAVIFLHGNATSSYLWRHVVPHIEPVARCIIPDLI
GMGKSGKSGNGSYRLLDHYKYLTAWFELLNLPKKIIFVGHDWGAALAFHYAYEHQDRI
KAIVHMESVVDVIESWDEWPDIEEDIALIKSEEGEKMVLENNFFVETVLPSKIMRKLEPE
EFAAYLEPFKEKGEVRRPTLSWPREIPLVKGGKPDVVQIVRNYNAYLRASDDLPKLFIE
SDPGFFSNAIVEGAKKFPNTEFVKVKGLHFLQEDAPDEMGKYIKSFVERVLKNEQ*

DNA sequence

ATGAAGACGATCATCGCCCTGAGCTACATCTTCTGCCTGGTATTCGCCGAACAAAA
GCTCATTTCCGAAGAGGATCTTGCCATGGAGACACTGTGTCTCAGGGCCTCCTTCT
GGCTCGCACTGGTAGGGTGTGTCATATCTGATAACCCTGAACGGTACAGTACCAAT
CTGAGTAATCATGTCGATGACTTTACAACGTTTCGGGGCACTGAGCTGAGCTTTCT
CGTGACTACGCACCAGCCCACCAATCTCGTCCTGCCTAGCAACGGGAGTATGCAC
AACTACTGCCCCCAGCAGACGAAAATAACAAGCGCCTTCAAGTATATCAACACCGT
GATATCCTGCACTATCTTCATAGTTGGCATGGTGGGGAACGCGACCCTGCTCCGC
ATTATTTATCAGAACAAGTGTATGAGAAACGGACCGAATGCACTTATCGCAAGTCT
GGCACTCGGAGACCTGATCTACGTCGTCATTGATCTGCCAATAAACGTGTTTAAGC
TCCTGGCCGGCCGCTGGCCCTTCGATCACAACGATTTTGGGGTGTTTCTTTGCAA
GCTCTTTCCTTTTTTGCAGAAATCATCCGTGGGCATCACCGTTCTCAATCTGTGTGC
GCTGTCCGTTGACCGATATCGCGCCGTCGCAAGCTGGTCCCGGGTCCAGGGAAT
CGGAATCCCACTGGTGACCGCTATCGAGATCGTGTCAATCTGGATACTGTCCTTTA
TCCTCGCGATTCCGGAGGCAATCGGGTTTGTGATGGTCCCTTTCGAGTATCGAGG
CGAGCAGCATAAAACCTGCATGTTGAATGCTACATCAAAGTTCATGGAGTTTTATCA
GGATGTGAAAGATTGGTGGCTTTTCGGATTCTATTTTTGCATGCCCTTGGTTTGTAC
CGCAGTGTGCTATACACTGATGATATTGCGGCTGAAATCTGTGCGCCTGCTGAGC
GGCAGCCGCGAAAAGGATCGGAATCTGCGCCGAATCACACGCCTCGTGCTGGTT
GTAGTCGCTGTCTTCGTGCTGTGCTGGTTCCCCCTGCATCTGAGCCGCATTCTCAA
GAAAACCGTCTACAACGAGATGGACAAAAATAGATGCGAATTGCTCTCCTTTTTGC
TTTTGATGGACTATATTGGGATTAACCTTGCCACCATGAATTCCTGTATCAACCCAA
TTGCTCTCTACTTTGTGTCAAAGAAATTCAAGAACTGTTTCCAAAGCTGTCTCTGTT
GCTGTTGCTACCAGTCTAAGAGTCTGATGACCTCAGTGCCCATGAATGGGACAAGT
ATCCAGTGGAAGAACCATGACCAGAATAACCACAACACAGACCGCTCATCCCATAA
GGATAGCATGAACTCTAGAGGGGGGATGGCTTCCAAGGTGTACGACCCCGAGCAA
CGCAAACGCATGATCACTGGGCCTCAGTGGTGGGCTCGCTGCAAGCAAATGAACG
TGCTGGACTCCTTCATCAACTACTATGATTCCGAGAAGCACGCCGAGAACGCCGT
GATTTTTCTGCATGGTAACGCTACCTCCAGCTACCTGTGGAGGCACGTCGTGCCTC
ACATCGAGCCCGTGGCTAGATGCATCATCCCTGATCTGATCGGAATGGGTAAGTC
CGGCAAGAGCGGGAATGGCTCATATCGCCTCCTGGATCACTACAAGTACCTCACC
GCTTGGTTCGAGCTGCTGAACCTTCCAAAGAAAATCATCTTTGTGGGCCACGACTG
GGGGGCTGCTCTGGCCTTTCACTACGCCTACGAGCACCAAGACAGGATCAAGGCC
ATCGTCCATATGGAGAGTGTCGTGGACGTGATCGAGTCCTGGGACGAGTGGCCTG
ACATCGAGGAGGATATCGCCCTGATCAAGAGCGAAGAGGGCGAGAAAATGGTGCT
TGAGAATAACTTCTTCGTCGAGACCGTGCTCCCAAGCAAGATCATGCGGAAACTG
GAGCCTGAGGAGTTCGCTGCCTACCTGGAGCCATTCAAGGAGAAGGGCGAGGTT
AGACGGCCTACCCTCTCCTGGCCTCGCGAGATCCCTCTCGTTAAGGGAGGCAAGC
CCGACGTCGTCCAGATTGTCCGCAACTACAACGCCTACCTTCGGGCCAGCGACGA
TCTGCCTAAGCTGTTCATCGAGTCCGACCCTGGGTTCTTTTCCAACGCTATTGTCG
AGGGAGCTAAGAAGTTCCCTAACACCGAGTTCGTGAAGGTGAAGGGCCTCCACTT

CCTCCAGGAGGACGCTCCAGATGAAATGGGTAAGTACATCAAGAGCTTCGTGGAG
CGCGTGCTGAAGAACGAGCAGTAA

3. Human HRH2

Protein sequence
MKTIIALSYIFCLVFAEQKLISEEDLAMAPNGTASSFCLDSTACKITITVVLAVLILITVAGN
VVVCLAVGLNRRLRNLTNCFIVSLAITDLLLGLLVLPFSAIYQLSCKWSFGKVFCNIYTSL
DVMLCTASILNLFMISLDRYCAVMDPLRYPVLVTPVRVAISLVLIWVISITLSFLSIHLGWN
SRNETSKGNHTTSKCKVQVNEVYGLVDGLVTFYLPLLIMC
VCYTLMILRLKSVRLLSGSREKDRNLRRITRLVLVVVAVFVICWFPYFTAFVYRGLRGD
DAINEVLEAIVLWLGYANSALNPILYAALNRDFRTGYQQLFCCRLANRNSHKTSLRSNA
SQLSRTQSREPRQQEEKPLKLQVWSGTEVTAPQGATDRSRGGMASKVYDPEQRKR
MITGPQWWARCKQMNVLDSFINYYDSEKHAENAVIFLHGNATSSYLWRHVVPHIEPVA
RCIIPDLIGMGKSGKSGNGSYRLLDHYKYLTAWFELLNLPKKIIFVGHDWGAALAFHYA
YEHQDRIKAIVHMESVVDVIESWDEWPDIEEDIALIKSEEGEKMVLENNFFVETVLPSKI
MRKLEPEEFAAYLEPFKEKGEVRRPTLSWPREIPLVKGGKPDVVQIVRNYNAYLRASD
DLPKLFIESDPGFFSNAIVEGAKKFPNTEFVKVKGLHFLQEDAPDEMGKYIKSFVERVL
KNEQ*

DNA sequence
ATGAAGACGATCATCGCCCTGAGCTACATCTTCTGCCTGGTATTCGCCGAACAAAA
GCTCATTTCCGAAGAGGATCTTGCCATGGCTCCTAATGGGACTGCCTCCTCCTTCT
GCCTTGACTCTACTGCCTGCAAAATAACTATTACAGTGGTACTCGCCGTCCTGATA
CTGATTACTGTTGCGGGTAATGTCGTAGTGTGTCTGGCCGTCGGCCTTAACCGGA
GACTGAGAAACCTTACTAACTGCTTCATTGTGAGCCTGGCGATCACTGATCTCCTC
CTGGGCCTCCTGGTGTTGCCCTTTAGTGCAATTTACCAGCTGTCATGTAAGTGGTC
CTTCGGAAAAGTATTCTGCAATATTTATACGAGCCTGGATGTGATGCTGTGCACGG
CCTCCATACTGAATCTGTTCATGATTTCCCTTGATCGGTATTGCGCCGTGATGGAT
CCCCTGCGGTACCCAGTCTTGGTGACCCCCGTTAGAGTGGCGATTAGTCTGGTCC
TCATTTGGGTTATCAGCATTACCCTCTCCTTCCTGAGCATCCATCTGGGCTGGAAC
TCCAGAAACGAGACCTCTAAGGGGAACCACACAACCAGTAAGTGCAAGGTGCAGG
TTAACGAGGTTTACGGGCTGGTAGATGGCCTTGTGACTTTTTACTTGCCCCTCCTG
ATTATGTGTGTGTGCTATACACTGATGATATTGCGGCTGAAATCTGTGCGCCTGCT
GAGCGGCAGCCGCGAAAAGGATCGGAATCTGCGCCGAATCACACGCCTCGTGCT
GGTTGTAGTCGCTGTCTTCGTGATCTGCTGGTTCCCTTACTTTACCGCCTTTGTGTA
CCGGGGCCTGCGAGGCGACGACGCTATTAACGAGGTCCTGGAGGCCATCGTCCT
GTGGCTGGGCTACGCCAACAGTGCTCTTAACCCAATCCTCTATGCGGCCCTCAAC
CGGGATTTTAGGACAGGGTACCAGCAGCTTTTTTGCTGCAGGCTTGCAAATCGGA
ATTCCCATAAGACATCCCTCCGGAGTAACGCCAGTCAGCTCAGCCGAACACAGAG
CAGGGAGCCGCGGCAGCAGGAGGAAAAACCTCTTAAGCTCCAGGTTTGGTCAGG
AACAGAGGTGACTGCCCCTCAAGGTGCTACCGACAGATCTAGAGGGGGGATGGC
TTCCAAGGTGTACGACCCCGAGCAACGCAAACGCATGATCACTGGGCCTCAGTGG

TGGGCTCGCTGCAAGCAAATGAACGTGCTGGACTCCTTCATCAACTACTATGATTC
CGAGAAGCACGCCGAGAACGCCGTGATTTTTCTGCATGGTAACGCTACCTCCAGC
TACCTGTGGAGGCACGTCGTGCCTCACATCGAGCCCGTGGCTAGATGCATCATCC
CTGATCTGATCGGAATGGGTAAGTCCGGCAAGAGCGGGAATGGCTCATATCGCCT
CCTGGATCACTACAAGTACCTCACCGCTTGGTTCGAGCTGCTGAACCTTCCAAAGA
AAATCATCTTTGTGGGCCACGACTGGGGGGCTGCTCTGGCCTTTCACTACGCCTA
CGAGCACCAAGACAGGATCAAGGCCATCGTCCATATGGAGAGTGTCGTGGACGTG
ATCGAGTCCTGGGACGAGTGGCCTGACATCGAGGAGGATATCGCCCTGATCAAGA
GCGAAGAGGGCGAGAAAATGGTGCTTGAGAATAACTTCTTCGTCGAGACCGTGCT
CCCAAGCAAGATCATGCGGAAACTGGAGCCTGAGGAGTTCGCTGCCTACCTGGAG
CCATTCAAGGAGAAGGGCGAGGTTAGACGGCCTACCCTCTCCTGGCCTCGCGAG
ATCCCTCTCGTTAAGGGAGGCAAGCCCGACGTCGTCCAGATTGTCCGCAACTACA
ACGCCTACCTTCGGGCCAGCGACGATCTGCCTAAGCTGTTCATCGAGTCCGACCC
TGGGTTCTTTTCCAACGCTATTGTCGAGGGAGCTAAGAAGTTCCCTAACACCGAGT
TCGTGAAGGTGAAGGGCCTCCACTTCCTCCAGGAGGACGCTCCAGATGAAATGGG
TAAGTACATCAAGAGCTTCGTGGAGCGCGTGCTGAAGAACGAGCAGTAA

4. Human 5-HT4R

Protein sequence
MKTIIALSYIFCLVFAEQKLISEEDLAMDKLDANVSSEEGFGSVEKVVLLTFLSTVILMAIL
GNLLVMVAVCWDRQLRKIKTNYFIVSLAFADLLVSVLVMPFGAIELVQDIWIYGEVFCLV
RTSLDVLLTTASIFHLCCISLDRYYAICCQPLVYRNKMTPLRIALMLGGCWVIPTFISFLPI
MQGWNNIGIIDLIEKRKFNQNSNSTYCVFMVNKPYAITCSVVAFYIPFLLMV
VCYTLMILRLKSVRLLSGSREKDRNLRRITRLVLVVVAVFVLCWAPFFVTNIVDPFIDYT
VPGQVWTAFLWLGYINSGLNPFLYAFLNKSFRRAFLIILCCDDERYRRPSILGQTVPCS
TTTINGSTHVLRDAVECGGQWESQCHPPATSPLVAAQPSDTSRGGMASKVYDPEQR
KRMITGPQWWARCKQMNVLDSFINYYDSEKHAENAVIFLHGNATSSYLWRHVVPHIEP
VARCIIPDLIGMGKSGKSGNGSYRLLDHYKYLTAWFELLNLPKKIIFVGHDWGAALAFH
YAYEHQDRIKAIVHMESVVDVIESWDEWPDIEEDIALIKSEEGEKMVLENNFFVETVLPS
KIMRKLEPEEFAAYLEPFKEKGEVRRPTLSWPREIPLVKGGKPDVVQIVRNYNAYLRAS
DDLPKLFIESDPGFFSNAIVEGAKKFPNTEFVKVKGLHFLQEDAPDEMGKYIKSFVERV
LKNEQ*

DNA sequence
ATGAAGACGATCATCGCCCTGAGCTACATCTTCTGCCTGGTATTCGCCGAACAAAA
GCTCATTTCCGAAGAGGATCTTGCCATGGATAAGCTGGATGCTAACGTTAGCAGTG
AAGAAGGATTTGGCAGTGTTGAGAAGGTGGTGCTGCTCACCTTCCTGTCCACTGT
GATCCTGATGGCAATCTTGGGTAACCTGCTCGTCATGGTAGCCGTTTGTTGGGATA
GGCAGCTGCGGAAAATCAAGACTAACTATTTCATAGTGTCTCTCGCCTTTGCAGAC
CTCCTGGTTAGTGTGCTGGTGATGCCATTCGGAGCCATCGAACTGGTGCAGGACA
TTTGGATCTATGGGGAAGTGTTCTGCCTGGTGAGAACTTCATTGGATGTTCTGCTG

ACTACTGCCAGCATTTTTCACCTTTGCTGTATTAGCCTGGACAGATATTACGCAATC
TGCTGCCAGCCACTGGTGTACAGGAATAAAATGACACCTTTGAGGATCGCCCTGAT
GCTCGGGGGTTGCTGGGTTATCCCCACCTTTATAAGTTTCTTGCCGATTATGCAGG
GTTGGAATAATATCGGAATAATAGACCTCATTGAGAAGCGCAAGTTCAATCAAAATT
CCAACTCTACCTACTGTGTATTCATGGTAAACAAACCGTACGCTATAACTTGCAGC
GTGGTCGCATTCTACATTCCCTTTCTCCTTATGGTGGTGTGCTATACACTGATGATA
TTGCGGCTGAAATCTGTGCGCCTGCTGAGCGGCAGCCGCGAAAAGGATCGGAAT
CTGCGCCGAATCACACGCCTCGTGCTGGTTGTAGTCGCTGTCTTCGTGTTGTGCT
GGGCACCATTTTTTGTAACCAACATCGTCGATCCTTTCATTGACTACACAGTGCCC
GGACAGGTGTGGACCGCCTTCCTCTGGTTGGGCTATATCAATAGCGGCCTGAATC
CTTTTCTCTATGCCTTCCTTAATAAATCCTTCAGGCGCGCATTTCTTATTATATTGTG
TTGCGATGATGAACGGTATAGGAGACCTTCTATCCTGGGACAGACCGTCCCTTGCT
CAACTACAACTATCAACGGTAGCACACATGTTCTGCGCGACGCCGTCGAGTGCGG
CGGTCAATGGGAGTCCCAGTGTCATCCTCCCGCTACCTCTCCACTCGTCGCGGCT
CAACCTTCAGATACTTCTAGAGGGGGGATGGCTTCCAAGGTGTACGACCCCGAGC
AACGCAAACGCATGATCACTGGGCCTCAGTGGTGGGCTCGCTGCAAGCAAATGAA
CGTGCTGGACTCCTTCATCAACTACTATGATTCCGAGAAGCACGCCGAGAACGCC
GTGATTTTTCTGCATGGTAACGCTACCTCCAGCTACCTGTGGAGGCACGTCGTGCC
TCACATCGAGCCCGTGGCTAGATGCATCATCCCTGATCTGATCGGAATGGGTAAG
TCCGGCAAGAGCGGGAATGGCTCATATCGCCTCCTGGATCACTACAAGTACCTCA
CCGCTTGGTTCGAGCTGCTGAACCTTCCAAAGAAAATCATCTTTGTGGGCCACGAC
TGGGGGGCTGCTCTGGCCTTTCACTACGCCTACGAGCACCAAGACAGGATCAAGG
CCATCGTCCATATGGAGAGTGTCGTGGACGTGATCGAGTCCTGGGACGAGTGGCC
TGACATCGAGGAGGATATCGCCCTGATCAAGAGCGAAGAGGGCGAGAAAATGGTG
CTTGAGAATAACTTCTTCGTCGAGACCGTGCTCCCAAGCAAGATCATGCGGAAACT
GGAGCCTGAGGAGTTCGCTGCCTACCTGGAGCCATTCAAGGAGAAGGGCGAGGT
TAGACGGCCTACCCTCTCCTGGCCTCGCGAGATCCCTCTCGTTAAGGGAGGCAAG
CCCGACGTCGTCCAGATTGTCCGCAACTACAACGCCTACCTTCGGGCCAGCGACG
ATCTGCCTAAGCTGTTCATCGAGTCCGACCCTGGGTTCTTTTCCAACGCTATTGTC
GAGGGAGCTAAGAAGTTCCCTAACACCGAGTTCGTGAAGGTGAAGGGCCTCCACT
TCCTCCAGGAGGACGCTCCAGATGAAATGGGTAAGTACATCAAGAGCTTCGTGGA
GCGCGTGCTGAAGAACGAGCAGTAA

5. Human 5-HT5AR

Protein sequence
MKTIIALSYIFCLVFAEQKLISEEDLAMDLPVNLTSFSLSTPSPLETNHSLGKDDLRPSSP
LLSVFGVLILTLLGFLVAATFAWNLLVLATILRVRTFHRVPHNLVASMAVSDVLVAALVM
PLSLVHELSGRRWQLGRRLCQLWIACDVLCCTASIWNVTAIALDRYWSITRHMEYTLR
TRKCVSNVMIALTWALSAVISLAPLLFGWGETYSEGSEECQVSREPSYAVFSTVGAFY
LPLCVVLFVYWKIYKALKSVRLLSGSREKDRNLRRAARMVGILIGVFVLCWIPFFLTELIS
PLCSCDIPAIWKSIFLWLGYSNSFFNPLIYTAFNKNYNSAFKNFFSRQHSRGGMASKVY
DPEQRKRMITGPQWWARCKQMNVLDSFINYYDSEKHAENAVIFLHGNATSSYLWRHV
VPHIEPVARCIIPDLIGMGKSGKSGNGSYRLLDHYKYLTAWFELLNLPKKIIFVGHDWGA

ALAFHYAYEHQDRIKAIVHMESVVDVIESWDEWPDIEEDIALIKSEEGEKMVLENNFFVE
TVLPSKIMRKLEPEEFAAYLEPFKEKGEVRRPTLSWPREIPLVKGGKPDVVQIVRNYNA
YLRASDDLPKLFIESDPGFFSNAIVEGAKKFPNTEFVKVKGLHFLQEDAPDEMGKYIKS
FVERVLKNEQ*

DNA sequence
ATGAAGACGATCATCGCCCTGAGCTACATCTTCTGCCTGGTATTCGCCGAACAAAA
GCTCATTTCCGAAGAGGATCTTGCCATGGACCTGCCCGTCAACTTGACGAGTTTCA
GTCTGTCCACTCCAAGTCCTCTTGAGACTAATCACTCTCTTGGAAAGGACGACTTG
AGGCCTTCCAGCCCTCTCCTCTCCGTGTTTGGGGTGCTGATTCTTACCCTGCTCGG
ATTTCTGGTAGCCGCTACTTTCGCATGGAACCTGTTGGTGCTGGCAACCATTCTCC
GCGTGCGGACATTCCACAGGGTTCCCCATAATCTTGTGGCATCCATGGCAGTGAG
TGACGTACTGGTAGCAGCACTGGTGATGCCTCTTTCTCTCGTCCATGAACTCAGCG
GGCGGAGATGGCAGCTTGGACGAAGACTTTGTCAGTTGTGGATTGCATGTGACGT
ACTTTGCTGTACTGCTTCCATTTGGAATGTCACCGCCATTGCCCTTGACCGCTACT
GGTCCATCACTCGCCATATGGAGTATACCTTGAGGACCCGAAAATGTGTCTCCAAT
GTTATGATCGCTCTGACCTGGGCCCTGAGCGCTGTGATCAGCCTTGCGCCGCTGC
TTTTCGGATGGGGAGAGACATATTCAGAGGGATCAGAGGAATGCCAGGTCAGTCG
GGAGCCTTCCTATGCGGTGTTTAGCACTGTCGGCGCTTTTTATCTCCCACTGTGTG
TCGTGCTGTTCGTCTATTGGAAAATTTATAAGGCTCTGAAATCTGTGCGCCTGCTG
AGCGGCAGCCGCGAAAAGGATCGGAATCTGCGCAGGGCAGCCcgtATGGTCGGGA
TCCTGATCGGTGTGTTTGTCCTCTGCTGGATACCCTTCTTCCTGACAGAACTTATCA
GCCCCCTCTGTAGCTGTGACATTCCCGCTATTTGGAAAAGCATCTTTCTGTGGCTT
GGATACAGTAACTCTTTTTTCAATCCTCTGATATACACAGCATTCAATAAAAACTACA
ACTCCGCATTCAAAAATTTCTTTTCACGGCAGCATTCTAGAGGGGGGATGGCTTCC
AAGGTGTACGACCCCGAGCAACGCAAACGCATGATCACTGGGCCTCAGTGGTGG
GCTCGCTGCAAGCAAATGAACGTGCTGGACTCCTTCATCAACTACTATGATTCCGA
GAAGCACGCCGAGAACGCCGTGATTTTTCTGCATGGTAACGCTACCTCCAGCTAC
CTGTGGAGGCACGTCGTGCCTCACATCGAGCCCGTGGCTAGATGCATCATCCCTG
ATCTGATCGGAATGGGTAAGTCCGGCAAGAGCGGGAATGGCTCATATCGCCTCCT
GGATCACTACAAGTACCTCACCGCTTGGTTCGAGCTGCTGAACCTTCCAAAGAAAA
TCATCTTTGTGGGCCACGACTGGGGGGCTGCTCTGGCCTTTCACTACGCCTACGA
GCACCAAGACAGGATCAAGGCCATCGTCCATATGGAGAGTGTCGTGGACGTGATC
GAGTCCTGGGACGAGTGGCCTGACATCGAGGAGGATATCGCCCTGATCAAGAGC
GAAGAGGGCGAGAAAATGGTGCTTGAGAATAACTTCTTCGTCGAGACCGTGCTCC
CAAGCAAGATCATGCGGAAACTGGAGCCTGAGGAGTTCGCTGCCTACCTGGAGCC
ATTCAAGGAGAAGGGCGAGGTTAGACGGCCTACCCTCTCCTGGCCTCGCGAGATC
CCTCTCGTTAAGGGAGGCAAGCCCGACGTCGTCCAGATTGTCCGCAACTACAACG
CCTACCTTCGGGCCAGCGACGATCTGCCTAAGCTGTTCATCGAGTCCGACCCTGG
GTTCTTTTCCAACGCTATTGTCGAGGGAGCTAAGAAGTTCCCTAACACCGAGTTCG
TGAAGGTGAAGGGCCTCCACTTCCTCCAGGAGGACGCTCCAGATGAAATGGGTAA
GTACATCAAGAGCTTCGTGGAGCGCGTGCTGAAGAACGAGCAGTAA

6. Human DRD1

Protein sequence

MKTIIALSYIFCLVFAEQKLISEEDLAMRTLNTSAMDGTGLVVERDFSVRILTACFLSLLIL
STLLGNTLVCAAVIRFRHLRSKVTNFFVISLAVSDLLVAVLVMPWKAVAEIAGFWPFGSF
CNIWVAFDIMCSTASILNLCVISVDRYWAISSPFRYERKMTPKAAFILISVAWTLSVLISFI
PVQLSWHKAKPTSPSDGNATSLAETIDNCDSSLSRTYAISSSVISFYIPVAIMIVTYTLMIL
RLKSVRLLSGSREKDRNLRRITRLVLVIMGVFVCCWLPFFILNCILPFCGSGETQPFCID
SNTFDVFVWFGWANSSLNPIIYAFNADFRKAFSTLLGCYRLCPATNNAIETVSINNNGA
AMFSSHHEPRGSISKECNLVYLIPHAVGSSEDLKKEEAAGIARPLEKLSPALSVILDYDT
DVSLEKIQPITQNGQHPTSRGGMASKVYDPEQRKRMITGPQWWARCKQMNVLDSFIN
YYDSEKHAENAVIFLHGNATSSYLWRHVVPHIEPVARCIIPDLIGMGKSGKSGNGSYRL
LDHYKYLTAWFELLNLPKKIIFVGHDWGAALAFHYAYEHQDRIKAIVHMESVVDVIESW
DEWPDIEEDIALIKSEEGEKMVLENNFFVETVLPSKIMRKLEPEEFAAYLEPFKEKGEVR
RPTLSWPREIPLVKGGKPDVVQIVRNYNAYLRASDDLPKLFIESDPGFFSNAIVEGAKK
FPNTEFVKVKGLHFLQEDAPDEMGKYIKSFVERVLKNEQ*

DNA sequence

ATGAAGACGATCATCGCCCTGAGCTACATCTTCTGCCTGGTATTCGCCGAACAAAA
GCTCATTTCCGAAGAGGATCTTGCCATGCGCACATTGAATACTTCAGCGATGGACG
GAACGGGCCTCGTGGTCGAACGGGACTTTAGCGTTAGAATTCTTACCGCTTGCTTC
CTGTCACTGCTTATCCTGTCTACTCTCCTGGGTAATACACTTGTGTGCGCCGCCGT
GATCAGATTCCGGCACTTGCGGTCCAAAGTGACTAACTTTTTTGTCATCTCACTGG
CCGTCTCTGATCTGCTGGTGGCTGTCCTCGTGATGCCATGGAAAGCAGTGGCCGA
GATCGCCGGGTTCTGGCCCTTCGGGTCATTTTGCAATATCTGGGTGGCATTCGAC
ATCATGTGTAGCACGGCCTCAATTCTCAATCTCTGTGTCATTAGTGTCGATAGGTAT
TGGGCCATCAGCTCTCCCTTCCGATACGAGCGGAAGATGACCCCAAAGGCCGCCT
TTATACTGATATCAGTCGCCTGGACCCTCAGCGTGCTGATTTCTTTCATCCCTGTG
CAATTGTCCTGGCACAAGGCTAAACCAACGAGTCCCAGCGACGGAAACGCCACTA
GTCTGGCCGAGACTATCGACAATTGTGACAGTAGCCTGTCCAGGACCTACGCTAT
CAGCAGTTCAGTGATCTCCTTTTACATTCCTGTCGCGATTATGATCGTGACCTACAC
CCTGATGATATTGCGGCTGAAATCTGTGCGCCTGCTGAGCGGCAGCCGCGAAAAG
GATCGGAATCTGCGCCGAATCACACGCCTGGTTCTGGTGATTATGGGCGTGTTTG
TGTGTTGCTGGCTCCCCTTCTTCATTTTGAATTGTATACTGCCTTTCTGCGGCAGTG
GCGAGACTCAGCCTTTCTGTATAGACTCAAACACTTTTGACGTCTTCGTTTGGTTCG
GTTGGGCCAATTCCTCCCTTAATCCTATTATATACGCATTTAATGCTGATTTTCGCA
AAGCATTCTCCACCCTGCTGGGCTGTTACCGGCTTTGTCCCGCCACCAATAACGCA
ATTGAGACCGTATCTATTAACAATAACGGCGCAGCTATGTTCTCCTCTCACCATGA
GCCAAGAGGTTCAATTAGCAAAGAGTGTAATCTTGTTTACTTGATACCTCACGCAG
TTGGGAGTTCAGAAGACCTTAAGAAAGAAGAAGCCGCCGGCATCGCTAGGCCCCT
CGAAAAACTGAGCCCTGCACTTTCTGTGATCCTGGATTATGACACTGATGTGAGCC
TGGAGAAAATTCAACCAATAACACAGAACGGTCAACATCCAACTTCTAGAGGGGGG

ATGGCTTCCAAGGTGTACGACCCCGAGCAACGCAAACGCATGATCACTGGGCCTC
AGTGGTGGGCTCGCTGCAAGCAAATGAACGTGCTGGACTCCTTCATCAACTACTAT
GATTCCGAGAAGCACGCCGAGAACGCCGTGATTTTTCTGCATGGTAACGCTACCT
CCAGCTACCTGTGGAGGCACGTCGTGCCTCACATCGAGCCCGTGGCTAGATGCAT
CATCCCTGATCTGATCGGAATGGGTAAGTCCGGCAAGAGCGGGAATGGCTCATAT
CGCCTCCTGGATCACTACAAGTACCTCACCGCTTGGTTCGAGCTGCTGAACCTTCC
AAAGAAAATCATCTTTGTGGGCCACGACTGGGGGGCTGCTCTGGCCTTTCACTAC
GCCTACGAGCACCAAGACAGGATCAAGGCCATCGTCCATATGGAGAGTGTCGTGG
ACGTGATCGAGTCCTGGGACGAGTGGCCTGACATCGAGGAGGATATCGCCCTGAT
CAAGAGCGAAGAGGGCGAGAAAATGGTGCTTGAGAATAACTTCTTCGTCGAGACC
GTGCTCCCAAGCAAGATCATGCGGAAACTGGAGCCTGAGGAGTTCGCTGCCTACC
TGGAGCCATTCAAGGAGAAGGGCGAGGTTAGACGGCCTACCCTCTCCTGGCCTC
GCGAGATCCCTCTCGTTAAGGGAGGCAAGCCCGACGTCGTCCAGATTGTCCGCAA
CTACAACGCCTACCTTCGGGCCAGCGACGATCTGCCTAAGCTGTTCATCGAGTCC
GACCCTGGGTTCTTTTCCAACGCTATTGTCGAGGGAGCTAAGAAGTTCCCTAACAC
CGAGTTCGTGAAGGTGAAGGGCCTCCACTTCCTCCAGGAGGACGCTCCAGATGAA
ATGGGTAAGTACATCAAGAGCTTCGTGGAGCGCGTGCTGAAGAACGAGCAGTAA

7. Rat Neurotensin 1 (NTS_1R)

Protein sequence
MKTIIALSYIFCLVFAEQKLISEEDLAMHLNSSVPQGTPGEPDAQPFSGPQSEMEATFLA
LSLSNGSGNTSESDTAGPNSDLDVNTDIYSKVLVTAIYLALFVVGTVGNSVTLFTLARKK
SLQSLQSTVHYHLGSLALSDLLILLLAMPVELYNFIWVHHPWAFGDAGCRGYYFLRDA
CTYATALNVASLSVERYLAICHPFKAKTLMSRSRTKKFISAIWLASALLAIPMLFTMGLQ
NRSADGTHPGGLVCTPIVDTATVKVVIQVNTFMSFLFPMLVISILYTLMILRLKSVRLLSG
SREKDRNLRRITRLVLAVVIAFVVCWLPYHVRRLMFCYISDEQWTTFLFDFYHYFYMLT
NALFYASSAINPILYNLVSANFRQVFLSTLACLCPGWRHRRKKRPTFSRKPNSMSSNH
AFSTSATRETLYSRGGMASKVYDPEQRKRMITGPQWWARCKQMNVLDSFINYYDSE
KHAENAVIFLHGNATSSYLWRHVVPHIEPVARCIIPDLIGMGKSGKSGNGSYRLLDHYK
YLTAWFELLNLPKKIIFVGHDWGAALAFHYAYEHQDRIKAIVHMESVVDVIESWDEWPD
IEEDIALIKSEEGEKMVLENNFFVETVLPSKIMRKLEPEEFAAYLEPFKEKGEVRRPTLS
WPREIPLVKGGKPDVVQIVRNYNAYLRASDDLPKLFIESDPGFFSNAIVEGAKKFPNTE
FVKVKGLHFLQEDAPDEMGKYIKSFVERVLKNEQ*

DNA sequence
ATGAAGACGATCATCGCCCTGAGCTACATCTTCTGCCTGGTATTCGCCGAACAAAA
GCTCATTTCCGAAGAGGATCTTGCCATGCACCTCAACAGCTCCGTGCCGCAGGGC
ACCCCTGGTGAACCCGATGCCCAGCCCTTTTCGGGACCACAGTCCGAAATGGAAG
CGACGTTCCTGGCGCTCAGTTTGAGCAATGGTTCTGGCAATACCTCGGAATCCGA
CACGGCAGGGCCCAACAGCGACCTGGACGTGAACACTGACATTTATTCCAAGGTG

CTGGTGACTGCTATATACCTGGCACTCTTCGTGGTGGGCACTGTGGGCAACTCCG
TGACActcTTCACTCTAGCGCGGAAGAAGTCACTGCAGAGCCTGCAGAGCACTGTG
CATTACCACCTGGGCAGCCTGGCACTGTCTGACCTGCTTATCCTTCTGCTGGCCAT
GCCCGTGGAGCTATACAACTTCATCTGGGTACACCATCCCTGGGCCTTTGGGGAC
GCTGGCTGCCGTGGCTACTATTTCCTGCGTGATGCCTGCACCTATGCCACAGCCC
TCAATGTAGCCAGCCTGAGTGTGGAGCGCTACTTGGCCATCTGCCATCCCTTCAA
GGCCAAGACCCTCATGTCCCGCAGCCGCACCAAGAAATTCATCAGTGCCATATGG
CTAGCTTCGGCGCTGCTGGCTATACCCATGCTTTTCACCATGGGCCTGCAGAACC
GCAGTgccGACGGCACGCACCCTGGCGGCCTGGTGTGCACACCCATTGTGGACAC
AGCCACTGTCAAGGTCGTCATCCAGGTTAACACCTTCATGTCCTTCCTGTTTCCCA
TGTTGGTCATCTCCATCCTATACACCCTGATGATATTGCGGCTGAAATCTGTGCGC
CTGCTGAGCGGCAGCCGCGAAAAGGATCGGAATCTGCGCCGAATCACACGCCTG
GTTCTGGCTGTGGTCATTGCCTTTGTGGTCTGCTGGCTGCCCTACCACGTGCGAC
GCCTGATGTTCTGCTATATCTCGGATGAACAGTGGACTACGTTCCTCTTCGATTTCT
ACCACTATTTCTACATGCTAACCAACGCTCTCTTCTACgccAGCTCCGCCATCAATC
CCATCCTCTACAACCTGGTCTCCGCCAACTTCCGCCAGGTCTTTCTGTCCACGCTG
GCCTGCCTTTGTCCTGGGTGGCGCCACCGCCGAAAGAAGAGGCCAACGTTCTCCA
GGAAGCCCAACAGCATGTCCAGCAACCATGCCTTTTCCACCAGCGCCACCCGGGA
GACCCTGTACTCTAGAGGGGGGATGGCTTCCAAGGTGTACGACCCCGAGCAACG
CAAACGCATGATCACTGGGCCTCAGTGGTGGGCTCGCTGCAAGCAAATGAACGTG
CTGGACTCCTTCATCAACTACTATGATTCCGAGAAGCACGCCGAGAACGCCGTGAT
TTTTCTGCATGGTAACGCTACCTCCAGCTACCTGTGGAGGCACGTCGTGCCTCACA
TCGAGCCCGTGGCTAGATGCATCATCCCTGATCTGATCGGAATGGGTAAGTCCGG
CAAGAGCGGGAATGGCTCATATCGCCTCCTGGATCACTACAAGTACCTCACCGCT
TGGTTCGAGCTGCTGAACCTTCCAAAGAAAATCATCTTTGTGGGCCACGACTGGG
GGGCTGCTCTGGCCTTTCACTACGCCTACGAGCACCAAGACAGGATCAAGGCCAT
CGTCCATATGGAGAGTGTCGTGGACGTGATCGAGTCCTGGGACGAGTGGCCTGA
CATCGAGGAGGATATCGCCCTGATCAAGAGCGAAGAGGGCGAGAAAATGGTGCTT
GAGAATAACTTCTTCGTCGAGACCGTGCTCCCAAGCAAGATCATGCGGAAACTGG
AGCCTGAGGAGTTCGCTGCCTACCTGGAGCCATTCAAGGAGAAGGGCGAGGTTA
GACGGCCTACCCTCTCCTGGCCTCGCGAGATCCCTCTCGTTAAGGGAGGCAAGCC
CGACGTCGTCCAGATTGTCCGCAACTACAACGCCTACCTTCGGGCCAGCGACGAT
CTGCCTAAGCTGTTCATCGAGTCCGACCCTGGGTTCTTTTCCAACGCTATTGTCGA
GGGAGCTAAGAAGTTCCCTAACACCGAGTTCGTGAAGGTGAAGGGCCTCCACTTC
CTCCAGGAGGACGCTCCAGATGAAATGGGTAAGTACATCAAGAGCTTCGTGGAGC
GCGTGCTGAAGAACGAGCAGTAA

8. GPR111

Protein sequence
MKTIIALSYIFCLVFAEQKLISEEDLAMTHILLLYYLVFLLPTESCRTLYQAASKSKEKVPA
RPHGVCDGVCTDYSQCTQPCPPDTQGNMGFSCRQKTWHKITDTCQTLNALNIFEEDS

RLVQPFEDNIKISVYTGKSETITDMLLQKCPTDLSCVIRNIQQSPWIPGNIAVIVQLLHNIS
TAIWTGVDEAKMQSYSTIANHILNSKSISNWTFIPDRNSSYILLHSVNSFARRLFIDKHPV
DISDVFIHTMGTTISGDNIGKNFTFSMRINDTSNEVTGRVLISRDELRKVPSPSQVISIAF
PTIGAILEASLLENVTVNGLVLSAILPKELKRISLIFEKISKSEERRTQCVGWHSVENRWD
QQACKMIQENSQQAVCKCRPSKLFTSFSILMSPHILESLILTYITYVGLGISICSLILCLSIE
VLVWSQVTKTEITYLRHVCIVNIAATLLMADVWFIVASFLSGPITHHKGCVAATFFVHFF
YLSVFFWMLAKALLILYGIMIVFHTLPKSVLVASLFSVGYGCPLAIAAITVAATEPGKGYL
RPEICWLNWDMTKALLAFVIPALAIVVVNLITVTLVYTLMILRLKSVRLLSGSREKDRNLR
RITRLVLLTPLLGLTWGFGVATVIDDRSLAFHIIFSLLNAFQGFFILVFGTILDPKIREALKG
SRGGMASKVYDPEQRKRMITGPQWWARCKQMNVLDSFINYYDSEKHAENAVIFLHG
NATSSYLWRHVVPHIEPVARCIIPDLIGMGKSGKSGNGSYRLLDHYKYLTAWFELLNLP
KKIIFVGHDWGAALAFHYAYEHQDRIKAIVHMESVVDVIESWDEWPDIEEDIALIKSEEG
EKMVLENNFFVETVLPSKIMRKLEPEEFAAYLEPFKEKGEVRRPTLSWPREIPLVKGGK
PDVVQIVRNYNAYLRASDDLPKLFIESDPGFFSNAIVEGAKKFPNTEFVKVKGLHFLQE
DAPDEMGKYIKSFVERVLKNEQ*

DNA sequence
ATGAAGACGATCATCGCCCTGAGCTACATCTTCTGCCTGGTATTCGCCGAACAAAA
GCTCATTTCCGAAGAGGATCTTGCCATGACACATATCCTTCTGTTGTATTATCTGGT
GTTTCTGCTGCCTACCGAGTCATGCAGGACTCTGTACCAAGCGGCAAGTAAGTCC
AAAGAGAAGGTCCCCGCACGCCCACACGGAGTTTGCGACGGTGTGTGTACAGACT
ACAGCCAGTGTACACAGCCATGCCCCCCTGACACACAGGGAAACATGGGGTTTAG
CTGTCGGCAAAAAACATGGCATAAGATCACCGACACTTGCCAGACCCTGAACGCTT
TGAACATATTCGAAGAAGATAGTAGGCTGGTGCAACCCTTCGAGGATAATATAAAA
ATCTCTGTTTACACTGGCAAATCCGAGACAATTACTGATATGCTGCTTCAAAAGTGT
CCTACGGACTTGAGTTGCGTGATTCGCAACATTCAACAGAGTCCCTGGATACCAG
GCAATATCGCCGTTATTGTGCAACTTTTGCATAACATTAGTACGGCCATCTGGACC
GGCGTCGACGAAGCGAAAATGCAGTCCTATAGTACGATCGCGAATCATATCCTGA
ATTCTAAAAGCATCTCCAATTGGACATTTATCCCTGATCGGAACTCCTCATATATCT
TGCTGCACAGCGTCAATTCATTCGCAAGGAGACTTTTTATTGATAAACATCCTGTCG
ATATCTCAGATGTTTTCATCCACACTATGGGTACCACTATTTCAGGTGACAACATCG
GGAAGAACTTCACTTTTTCAATGCGCATCAACGACACATCTAATGAAGTAACTGGC
CGCGTATTGATTAGCCGAGATGAACTCAGGAAGGTCCCCTCTCCATCTCAGGTTAT
TTCCATCGCGTTTCCAACCATTGGAGCAATACTGGAAGCATCTCTCCTTGAAAATGT
TACCGTCAATGGTCTGGTCCTTTCTGCCATACTGCCAAAAGAGCTGAAAAGAATTT
CCCTCATCTTTGAAAAGATTAGTAAATCAGAGGAAAGACGAACTCAGTGCGTAGGT
TGGCACTCAGTGGAGAATAGGTGGGATCAACAGGCATGTAAGATGATACAGGAAA
ACAGCCAGCAAGCAGTGTGCAAATGTCGCCCCTCTAAACTCTTCACCTCTTTCTCC
ATCCTGATGTCTCCTCACATACTGGAGTCCCTCATCTTGACTTACATTACATATGTG
GGCCTGGGGATTTCTATATGTTCCCTGATCCTCTGTCTTTCTATTGAAGTTCTTGTT
TGGTCACAGGTTACAAAGACGGAGATTACTTACCTGAGACATGTTTGCATAGTCAA
CATCGCAGCCACATTGCTGATGGCTGATGTATGGTTTATCGTGGCCAGTTTTCTGT

CTGGACCTATAACACACCACAAAGGATGCGTTGCCGCAACCTTCTTCGTTCACTTC
TTTTATCTGTCTGTTTTTTTTTGGATGCTTGCCAAAGCCCTGTTGATCCTCTACGGA
ATAATGATCGTATTCCACACCCTCCCTAAAAGCGTACTTGTGGCCTCTCTGTTTTCA
GTGGGCTACGGTTGTCCCCTGGCCATAGCTGCAATCACCGTGGCGGCCACAGAA
CCAGGGAAAGGATATCTCCGGCCAGAAATCTGCTGGCTGAACTGGGACATGACTA
AGGCCCTGCTTGCCTTTGTCATCCCTGCGCTTGCTATAGTGGTAGTCAACTTGATC
ACGGTGACACTGGTGTATACACTGATGATATTGCGGCTGAAATCTGTGCGCCTGCT
GAGCGGCAGCCGCGAAAAGGATCGGAATCTGCGCCGAATCACACGCCTCGTGCT
GCTGACCCCCCTCCTGGGATTGACCTGGGGATTCGGCGTAGCCACCGTCATAGAC
GACAGAAGCTTGGCCTTTCATATCATTTTCAGCTTGCTGAATGCATTCCAAGGCTTT
TTTATCCTGGTCTTTGGAACAATTCTGGACCCGAAGATCAGAGAGGCTCTGAAGGG
GTCTAGAGGGGGGATGGCTTCCAAGGTGTACGACCCCGAGCAACGCAAACGCAT
GATCACTGGGCCTCAGTGGTGGGCTCGCTGCAAGCAAATGAACGTGCTGGACTCC
TTCATCAACTACTATGATTCCGAGAAGCACGCCGAGAACGCCGTGATTTTTCTGCA
TGGTAACGCTACCTCCAGCTACCTGTGGAGGCACGTCGTGCCTCACATCGAGCCC
GTGGCTAGATGCATCATCCCTGATCTGATCGGAATGGGTAAGTCCGGCAAGAGCG
GGAATGGCTCATATCGCCTCCTGGATCACTACAAGTACCTCACCGCTTGGTTCGAG
CTGCTGAACCTTCCAAAGAAAATCATCTTTGTGGGCCACGACTGGGGGGCTGCTC
TGGCCTTTCACTACGCCTACGAGCACCAAGACAGGATCAAGGCCATCGTCCATAT
GGAGAGTGTCGTGGACGTGATCGAGTCCTGGGACGAGTGGCCTGACATCGAGGA
GGATATCGCCCTGATCAAGAGCGAAGAGGGCGAGAAAATGGTGCTTGAGAATAAC
TTCTTCGTCGAGACCGTGCTCCCAAGCAAGATCATGCGGAAACTGGAGCCTGAGG
AGTTCGCTGCCTACCTGGAGCCATTCAAGGAGAAGGGCGAGGTTAGACGGCCTAC
CCTCTCCTGGCCTCGCGAGATCCCTCTCGTTAAGGGAGGCAAGCCCGACGTCGTC
CAGATTGTCCGCAACTACAACGCCTACCTTCGGGCCAGCGACGATCTGCCTAAGC
TGTTCATCGAGTCCGACCCTGGGTTCTTTTCCAACGCTATTGTCGAGGGAGCTAAG
AAGTTCCCTAACACCGAGTTCGTGAAGGTGAAGGGCCTCCACTTCCTCCAGGAGG
ACGCTCCAGATGAAATGGGTAAGTACATCAAGAGCTTCGTGGAGCGCGTGCTGAA
GAACGAGCAGTAA

9. GPR149

Protein sequence
MKTIIALSYIFCLVFAEQKLISEEDLA
MSLFLSNLSTNDSSLWKENHNSTDLLNPPGTLNIYLFCLTCLMTFAALVGSIYSLISLLK
MQNRTVVSMLVASWSVDDLMSVLSVTIFMFLQWPNEVPGYFQFLCTTSALMYLCQGL
SSNLKATLLVSYNFYTMHRGVGSQTASRRSGQVLGVVLTVWAASLLLSALPLCGWGA
FVRTPWGCLVDCSSSYVLFLSIVYALAFGLLVGLSVPLTHRLLCVTLVYTLMILRLKSVR
LLSGSREKDRNLRRITRLVLILALTKVVLWLPMMMHMVVQNVVGFQSLPLETFSFLLTL
LATTVTPVFVLSKRWTHLPCGCIINCRQNAYAVASDGKKIKRKGFEFNLSFQKSYGIYKI
AHEDYYDDDENSIFYHNLMNSECETTKDPQRDNRNIFNAIKVEISTTPSLDSSTQRGIN
KCTNTDITEAKQDSNNKKDAFSDKTGGDINYEETTFSEGPERRLSHEESQKPDLSDWE
WCRSKSERTPRQRSGYALAIPLCAFQGTVSLHAPTGKTLSLSTYEVSAEGQKITPASK
KIEVYRSKSVGHEPNSEDSSSTFVDTSVKIHLEVLEICDNEEALDTVSIISNISQSSTQVR

SPSLRYSRKENRFVSCDLGETASYSLFLPTSNPDGDINISIPDTVEAHRQNSKRQHQER
DGYQEEIQLLNKAYRKREEESKGSSRGGMASKVYDPEQRKRMITGPQWWARCKQM
NVLDSFINYYDSEKHAENAVIFLHGNATSSYLWRHVVPHIEPVARCIIPDLIGMGKSGKS
GNGSYRLLDHYKYLTAWFELLNLPKKIIFVGHDWGAALAFHYAYEHQDRIKAIVHMESV
VDVIESWDEWPDIEEDIALIKSEEGEKMVLENNFFVETVLPSKIMRKLEPEEFAAYLEPF
KEKGEVRRPTLSWPREIPLVKGGKPDVVQIVRNYNAYLRASDDLPKLFIESDPGFFSNA
IVEGAKKFPNTEFVKVKGLHFLQEDAPDEMGKYIKSFVERVLKNEQ*

DNA sequence
ATGAAGACGATCATCGCCCTGAGCTACATCTTCTGCCTGGTATTCGCCGAACAAAA
GCTCATTTCCGAAGAGGATCTTGCCATGAGTTTGTTCCTGTCCAATCTCTCCACTAA
CGATTCAAGTCTCTGGAAAGAGAACCATAACTCTACAGACCTGCTTAATCCTCCCG
GCACACTCAATATCTACCTTTTTTGCCTGACCTGCCTGATGACTTTTGCAGCCCTCG
TGGGCTCCATCTATAGCCTCATCAGCCTGCTTAAGATGCAGAATCGAACCGTGGTC
AGCATGCTGGTTGCTTCATGGTCAGTCGATGACCTCATGTCCGTCCTCTCTGTAAC
CATTTTTATGTTTCTGCAGTGGCCCAACGAGGTCCCCGGCTATTTCCAGTTCTTGT
GCACCACTTCCGCTCTGATGTACCTGTGTCAGGGCCTCAGCTCTAATCTCAAGGCC
ACCCTGCTGGTATCTTATAACTTCTATACTATGCACAGGGGCGTAGGTAGCCAGAC
AGCCTCCCGGAGGAGTGGCCAAGTGCTCGGAGTAGTACTTACCGTATGGGCCGC
TTCCCTCCTTTTGTCCGCACTGCCACTGTGTGGCTGGGGAGCCTTCGTGAGAACT
CCTTGGGGCTGCTTGGTGGACTGCTCTTCCAGTTATGTGTTGTTTCTGAGTATCGT
GTATGCTCTGGCTTTTGGGTTGCTTGTGGGTTTGTCCGTGCCCCTGACGCATAGGT
TGTTGTGCTATACACTGATGATATTGCGGCTGAAATCTGTGCGCCTGCTGAGCGGC
AGCCGCGAAAAGGATCGGAATCTGCGCCGAATCACACGCCTCGTGCTG
ATTTTGGCACTCACTAAAGTGGTTCTCTGGCTGCCCATGATGATGCATATGGTGGT
ACAGAACGTGGTAGGTTTTCAGAGTCTGCCACTTGAGACCTTCTCCTTTTTGCTGA
CCCTGCTGGCCACAACTGTCACTCCAGTGTTCGTACTTAGCAAGCGATGGACGCA
TCTGCCTTGCGGCTGCATTATTAATTGCAGGCAAAATGCGTATGCCGTCGCGTCCG
ACGGGAAAAAGATAAAGCGAAAGGGGTTCGAGTTTAACCTTTCTTTCCAGAAGTCT
TACGGGATCTACAAAATTGCGCACGAAGACTATTACGACGATGATGAAAACTCCAT
CTTCTACCACAATCTGATGAACAGCGAATGCGAGACAACAAAGGACCCCCAGCGA
GATAATAGAAATATTTTTAACGCCATTAAGGTCGAAATCAGTACCACCCCTAGCCTG
GATTCTTCTACCCAACGCGGGATTAATAAATGCACAAACACTGATATAACAGAGGC
TAAGCAGGACTCTAACAACAAAAAGGATGCCTTCAGCGATAAAACAGGGGGGGAC
ATAAATTACGAGGAGACTACATTCTCAGAGGGGCCTGAAAGACGGCTGTCACACG
AGGAGTCACAAAAACCAGACCTTAGCGACTGGGAATGGTGCAGGTCTAAGTCAGA
GCGGACTCCACGACAACGGAGCGGTTACGCCCTCGCGATTCCCCTGTGCGCCTTC
CAGGGCACTGTGAGCCTGCATGCCCCAACAGGGAAGACGCTCTCATTGAGTACCT
ATGAGGTTAGCGCCGAGGGCCAGAAAATAACTCCTGCTAGCAAGAAGATAGAGGT
GTATAGGTCAAAGTCCGTCGGCCATGAACCCAACAGCGAAGATTCCTCTTCCACCT
TTGTCGATACATCCGTGAAGATACATCTGGAGGTGCTGGAGATTTGCGACAATGAG
GAAGCGCTTGATACAGTTAGTATAATTAGTAACATCTCCCAGTCTTCCACCCAGGT

AAGATCACCCTCATTGAGGTATTCCCGCAAAGAGAACCGATTTGTTAGCTGCGACC
TTGGAGAAACAGCCTCTTATTCCTTGTTTCTCCCGACCTCAAACCCAGACGGGGAC
ATCAATATAAGCATCCCAGATACGGTTGAAGCTCACAGACAGAACTCCAAGCGGCA
GCACCAGGAAAGGGATGGCTATCAGGAAGAAATCCAGCTGCTCAATAAAGCCTAC
CGGAAACGGGAAGAGGAGTCCAAAGGGAGCTCTAGAGGGGGGATGGCTTCCAAG
GTGTACGACCCCGAGCAACGCAAACGCATGATCACTGGGCCTCAGTGGTGGGCT
CGCTGCAAGCAAATGAACGTGCTGGACTCCTTCATCAACTACTATGATTCCGAGAA
GCACGCCGAGAACGCCGTGATTTTTCTGCATGGTAACGCTACCTCCAGCTACCTGT
GGAGGCACGTCGTGCCTCACATCGAGCCCGTGGCTAGATGCATCATCCCTGATCT
GATCGGAATGGGTAAGTCCGGCAAGAGCGGGAATGGCTCATATCGCCTCCTGGAT
CACTACAAGTACCTCACCGCTTGGTTCGAGCTGCTGAACCTTCCAAAGAAAATCAT
CTTTGTGGGCCACGACTGGGGGGCTGCTCTGGCCTTTCACTACGCCTACGAGCAC
CAAGACAGGATCAAGGCCATCGTCCATATGGAGAGTGTCGTGGACGTGATCGAGT
CCTGGGACGAGTGGCCTGACATCGAGGAGGATATCGCCCTGATCAAGAGCGAAG
AGGGCGAGAAAATGGTGCTTGAGAATAACTTCTTCGTCGAGACCGTGCTCCCAAG
CAAGATCATGCGGAAACTGGAGCCTGAGGAGTTCGCTGCCTACCTGGAGCCATTC
AAGGAGAAGGGCGAGGTTAGACGGCCTACCCTCTCCTGGCCTCGCGAGATCCCT
CTCGTTAAGGGAGGCAAGCCCGACGTCGTCCAGATTGTCCGCAACTACAACGCCT
ACCTTCGGGCCAGCGACGATCTGCCTAAGCTGTTCATCGAGTCCGACCCTGGGTT
CTTTTCCAACGCTATTGTCGAGGGAGCTAAGAAGTTCCCTAACACCGAGTTCGTGA
AGGTGAAGGGCCTCCACTTCCTCCAGGAGGACGCTCCAGATGAAATGGGTAAGTA
CATCAAGAGCTTCGTGGAGCGCGTGCTGAAGAACGAGCAGTAA

10. GPR151

Protein sequence

MKTIIALSYIFCLVFAEQKLISEEDLAMLAAAFADSNSSSMNVSFAHLHFAGGYLPSDSQ
DWRTIIPALLVAVCLVGFVGNLCVIGILLHNAWKGKPSMIHSLILNLSLADLSLLLFSAPIR
ATAYSKSVWDLGWFVCKSSDWFIHTCMAAKSLTIVVVAKVCFMYASDPAKQVSIHNYT
IWSVLVAIWTVASLLPLPEWFFSTIRHHEGVEMCLVDVPAVAEEFMSMFGKLYPLLAFG
LPLFFASFYVTLVYTLMILRLKSVRLLSGSREKDRNLRRITRLVLSIAIISALLWLPEWVA
WLWVWHLKAAGPAPPQGFIALSQVLMFSISSANPLIFLVMSEEFREGLKGVWKWMITK
KPPTVSESQETPAGNSEGLPDKVPSPESPASIPEKEKPSSPSSGKGKTEKAEIPILPDV
EQFWHERDTVPSVQDNDPIPWEHEDQETGEGVKSRGGMASKVYDPEQRKRMITGP
QWWARCKQMNVLDSFINYYDSEKHAENAVIFLHGNATSSYLWRHVVPHIEPVARCIIP
DLIGMGKSGKSGNGSYRLLDHYKYLTAWFELLNLPKKIIFVGHDWGAALAFHYAYEHQ
DRIKAIVHMESVVDVIESWDEWPDIEEDIALIKSEEGEKMVLENNFFVETVLPSKIMRKL
EPEEFAAYLEPFKEKGEVRRPTLSWPREIPLVKGGKPDVVQIVRNYNAYLRASDDLPK
LFIESDPGFFSNAIVEGAKKFPNTEFVKVKGLHFLQEDAPDEMGKYIKSFVERVLKNEQ*

DNA sequence

ATGAAGACGATCATCGCCCTGAGCTACATCTTCTGCCTGGTATTCGCCGAACAAAA
GCTCATTTCCGAAGAGGATCTTGCCATGTTGGCCGCCGCATTTGCCGATTCAAATA
GTTCCAGTATGAACGTTTCCTTCGCTCACCTTCATTTTGCTGGTGGCTACTTGCCCA
GCGACAGCCAAGATTGGCGGACAATTATTCCCGCTCTCCTGGTTGCAGTATGTTTG
GTGGGTTTTGTGGGGAATCTTTGCGTCATCGGTATACTTCTGCACAACGCCTGGAA
AGGCAAGCCATCCATGATCCACAGCCTCATTCTTAATCTGTCTCTGGCAGACCTCT
CCCTCTTGCTGTTCAGCGCCCCGATACGGGCGACAGCGTACAGTAAGAGTGTCTG
GGACTTGGGCTGGTTTGTATGCAAAAGTTCCGATTGGTTTATCCACACCTGTATGG
CCGCAAAGTCCCTGACAATCGTGGTCGTGGCAAAGGTTTGCTTTATGTATGCCAGT
GACCCTGCTAAGCAAGTGTCTATTCATAATTATACTATATGGTCAGTGCTGGTGGC
CATCTGGACAGTGGCCTCTCTCCTGCCCCTGCCAGAATGGTTTTTTCCACCATCC
GCCACCACGAGGGGGTTGAGATGTGCTTGGTGGACGTGCCGGCCGTGGCTGAGG
AGTTCATGTCTATGTTTGGAAAGTTGTACCCATTGCTTGCATTCGGGCTGCCGCTC
TTTTTCGCATCATTCTACTATACACTGATGATATTGCGGCTGAAATCTGTGCGCCTG
CTGAGCGGCAGCCGCGAAAAGGATCGGAATCTGCGCCGAATCACACGCCTCGTG
CTGTCTATTGCCATCATCTCCGCGCTGCTGTGGCTGCCAGAATGGGTGGCTTGGC
TCTGGGTATGGCATCTCAAAGCAGCCGGCCCTGCCCCACCTCAGGGCTTTATCGC
ACTCTCCCAGGTCCTGATGTTCTCTATCTCCAGTGCCAATCCCCTGATCTTCCTCG
TGATGTCCGAAGAATTTAGAGAGGGTCTCAAAGGAGTCTGGAAGTGGATGATAACA
AAAAAGCCCCCAACAGTGAGCGAGTCACAGGAGACACCGGCCGGAAATAGTGAA
GGTCTTCCGGATAAGGTACCTAGTCCCGAAAGCCCTGCCTCCATCCCGGAGAAGG
AAAAACCGAGCTCCCCCTCCTCCGGAAAAGGAAAGACAGAAAAGGCAGAGATACC
GATTCTTCCAGACGTAGAGCAATTCTGGCACGAGCGCGACACCGTGCCCAGCGTG
CAGGATAATGATCCAATCCCTTGGGAGCATGAGGACCAAGAAACTGGAGAAGGAG
TGAAGTCTAGAGGGGGGATGGCTTCCAAGGTGTACGACCCCGAGCAACGCAAAC
GCATGATCACTGGGCCTCAGTGGTGGGCTCGCTGCAAGCAAATGAACGTGCTGGA
CTCCTTCATCAACTACTATGATTCCGAGAAGCACGCCGAGAACGCCGTGATTTTTC
TGCATGGTAACGCTACCTCCAGCTACCTGTGGAGGCACGTCGTGCCTCACATCGA
GCCCGTGGCTAGATGCATCATCCCTGATCTGATCGGAATGGGTAAGTCCGGCAAG
AGCGGGAATGGCTCATATCGCCTCCTGGATCACTACAAGTACCTCACCGCTTGGTT
CGAGCTGCTGAACCTTCCAAAGAAAATCATCTTTGTGGGCCACGACTGGGGGGCT
GCTCTGGCCTTTCACTACGCCTACGAGCACCAAGACAGGATCAAGGCCATCGTCC
ATATGGAGAGTGTCGTGGACGTGATCGAGTCCTGGGACGAGTGGCCTGACATCGA
GGAGGATATCGCCCTGATCAAGAGCGAAGAGGGCGAGAAAATGGTGCTTGAGAAT
AACTTCTTCGTCGAGACCGTGCTCCCAAGCAAGATCATGCGGAAACTGGAGCCTG
AGGAGTTCGCTGCCTACCTGGAGCCATTCAAGGAGAAGGGCGAGGTTAGACGGC
CTACCCTCTCCTGGCCTCGCGAGATCCCTCTCGTTAAGGGAGGCAAGCCCGACGT
CGTCCAGATTGTCCGCAACTACAACGCCTACCTTCGGGCCAGCGACGATCTGCCT
AAGCTGTTCATCGAGTCCGACCCTGGGTTCTTTTCCAACGCTATTGTCGAGGGAGC
TAAGAAGTTCCCTAACACCGAGTTCGTGAAGGTGAAGGGCCTCCACTTCCTCCAG
GAGGACGCTCCAGATGAAATGGGTAAGTACATCAAGAGCTTCGTGGAGCGCGTGC
TGAAGAACGAGCAGTAA

KOR-L253A-REV	GATTTCGCCCGCAATATCATCAGTGTATAGCACACTATGATAATCAGC
KOR-L253A-FOR	GCGGGCGAAATCTGTGCGCCTGCTGAGC
KOR-R257A-REV	GCAGGGCCACAGATTTCAGCCGCAATATCATCAGTG
KOR-R257A-FOR	GTGGCCCTGCTGAGCGGCAGCCGCG
KOR-R263A-REV	CTTTTCGGCGCTGCCGCTCAGCAGGC
KOR-R263A-FOR	CAGCGCCGAAAAGGATCGGAATCTGCGCCG
KOR-D266A-FOR	CCGGGCCTTTTCGCGGCTGCCGCTCAG
KOR-R266A-FOR	GAAAAGGCCCGGAATCTGCGCCGAATCACAC
KORD266L-REV	GATTCCGCAGCTTTTCGCGGCTGCCGCTC
KORD266L-FOR	GCTGCGGAATCTGCGCCGAATCACAC
KOR-R270A-REV	GATTCGGGCCAGATTCCGATCCTTTTCGCGGC
KOR-R270A-FOR	CTGGCCCGAATCACACGCCTCGTGCTG
KORR270M-REV	GATTCGCATCAGATTCCGATCCTTTTCGCGGC
KORR270M-FOR	CTGATGCGAATCACACGCCTCGTGCTG
KOR-R271A-REV	GTGATGGCGCGCAGATTCCGATCCTTTTCGC
KOR-R271A-FOR	GCGCGCCATCACACGCCTCGTGCTGGTTG
KOR-R274A-REV	CACGAGGGCTGTGATTCGGCGCAGATTCCGATC
KOR-R274A-FOR	CACAGCCCTCGTGCTGGTTGTAGTCGCTGTC
Nb6F31A-REV	GAGTCTGGCGATGGTTCCAGAGGCTGCACAGGAG
Nb6F31A-FOR	CCATCGCCAGACTCTATGATATGGGCTGGTACCGTC
Nb6F34A-REV	CATATCAGCGAGTCTGAAGATGGTTCCAGAGGCTGC
Nb6F34A-FOR	CAGACTCGCTGATATGGGCTGGTACCGTCGGGTTTC
Nb6R102A-REV	CGGTAGCGTACTCTGCATTACAGTAATAGACGGCTGTGTC
Nb6R102A-FOR	GAGTACGCTACCGGTATTTGGGAGGAACTATTAGACGG
Nb6W106A-REV	GTTCCTCCGCAATACCGGTACGGTACTCTGCATTACAGTAATAG
Nb6W106A-FOR	GTATTGCGGAGGAACTATTAGACGGCTGGGGC
Nb6-I105A-REV	CCTCCCAAGCACCGGTACGGTACTCTGCATTACAGTAATAG
Nb6-I105A-FOR	CCGGTGCTTGGGAGGAACTATTAGACGGCTGGG
Nb6-E107A-REV	CTAATAGTTCCGCCCAAATACCGGTACGGTACTCTGCATTAC
Nb6-E107A-FOR	GTATTTGGGCGGAACTATTAGACGGCTGGGGCCAG
Nb6-E108A-REV	CTAATAGTGCCTCCCAAATACCGGTACGGTACTCTGC
Nb6-E108A-FOR	GAGGCACTATTAGACGGCTGGGGCCAGGG
KORICL3-For	GTGTGCTATACACTGATGATATTGCGGCTGAAATC
KORICL3-Rev	CACGAAGACAGCGACTACAACCAGC
5HT2A-Rev	CATCAGTGTATAGCACACCACCATAATGGTAAGAGGAATGAAGAAGGACACG
5HT2A-For	GTCGCTGTCTTCGTGGTGATGTGGTGTCCATTCTTTATAACGAACATCATG
EDRNA-FOR	GTATAGCACACTGCGGTACAAACCAAGGGCATGC
KORICL3-FOR	GCCCTTGGTTTGTACCGCAGTGTGCTATACACTGATGATATTGCGGCTG
KORICL3-REV	GGGGGAACCAGCACAGCACGAAGACAGCGACTACAACCAGC
EDRNA-REV	GTCTTCGTGCTGTGCTGGTTCCCCCTGCATC
5HTR4-REV	CAATATCATCAGTGTATAGCACACCACCATAAGGAGAAAGGGAATGTAGAATGCG
KORICL3-FOR	GTGTGCTATACACTGATGATATTGCGGC
KORICL3-REV	CACGAAGACAGCGACTACAACCAG
5HTR4-FOR	GTTGTAGTCGCTGTCTTCGTGTTGTGCTGGGCACCATTTTTTGTAACC
HRH2-Rev	CAGTGTATAGCACACACACATAATCAGGAGGGGCAAGTAAAAAGTC
HRH2-For	GTCGCTGTCTTCGTGATCTGCTGGTTCCCTTACTTTACCGCC
KORICL3-For	GTGTGCTATACACTGATGATATTGCGGCTGAAATC
KORICL3-Rev	CACGAAGACAGCGACTACAACCAGC
5HTR4bb-REV-	CAATATCATCAGTGTATAGCACACCACCATAAGGAGAAAGGGAATGTAGAATGCG
KORinsert-FOR	GTGTGCTATACACTGATGATATTGCGGC
KORinsert-REV	CACGAAGACAGCGACTACAACCAG
5HTR4bb-FOR	GTTGTAGTCGCTGTCTTCGTGTTGTGCTGGGCACCATTTTTTGTAACC
5HT5A-KOR-Rev	CTTTTCGCGGCTGCCGCTCAGCAGGCGCACAGATTTCAGAGCCTTATAAATTTTCCAATAGACGAACAGCACG
5HT5A-KOR-For	GCGGCAGCCGCGAAAAGGATCGGAATCTGCGCAGGGCAGCCcgtATGGTCGGGATC
DRD1-KOR-Rev	GATCCTTTTCGCGGCTGCCGCTCAGCAGGCGCACAGATTTCAGCCGCAATATCATCAGTGTGTAGGTCACGATCATAA
DRD1-KOR-For	TGTGCGCCTGCTGAGCGGCAGCCGCGAAAAGGATCGGAATCTGCGCCGAATCACACGCACCCTCTCTGTGATTATGGG
NTS1R-KOR-Rev	GATCCTTTTCGCGGCTGCCGCTCAGCAGGCGCACAGATTTCAGCCGCAATATCATCAGGGTGTATAGGATGGAGATGACCAACATGGG
NTS1R-KOR-For	CGCCTGCTGAGCGGCAGCCGCGAAAAGGATCGGAATCTGCGCCGAATCACACGCCTGGTGCTGGCTGTGGTCATTGCCTTTGTGGTC
GPR111-KOR-Rev	CTTTTCGCGGCTGCCGCTCAGCAGGCGCACAGATTTCAGCCGCAATATCATCAGTGTATACACCAGTGTCACCGTGATCAAGTTG
GPR111-KOR-For	GGCAGCCGCGAAAAGGATCGGAATCTGCGCCGAATCACACGCCTCGTGCTGCTGACCCCCCTCCTGGGATTGAC
GPR149-KOR-Rev	CGCGGCTGCCGCTCAGCAGGCGCACAGATTTCAGCCGCAATATCATCAGTGTATAGCACAACAACCTATGCGTCAGGGGC
GPR149-KOR-For	CTGAGCGGCAGCCGCGAAAAGGATCGGAATCTGCGCCGAATCACACGCCTCGTGCTGATTTTGGCACTCACTAAAGTGGTTCTCTGG
GPR151-KOR-Rev	CGCGGCTGCCGCTCAGCAGGCGCACAGATTTCAGCCGCAATATCATCAGTGTATAGTAGAATGATGCGAAAAAGAGCGGCAGC
GPR151-KOR-For	CTGAGCGGCAGCCGCGAAAAGGATCGGAATCTGCGCCGAATCACACGCCTCGTGCTGTCTATTGCCATCATCTCCGCGCTG
